# A Semi-Automated Workflow for Quantitative Analysis of Right Ventricular Outflow Tract Geometry Based on 3D Point-Cloud Models

**DOI:** 10.3390/s26217037

**Published:** 2026-11-06

**Authors:** Katarzyna Bobkowska, Agata Kaczyńska-Miotk, Adam Kosiński

**Affiliations:** 1 Faculty of Civil and Environmental Engineering, Gdańsk University of Technology, Narutowicza 11/12, 80-233 Gdańsk, Poland; katarzyna.bobkowska@pg.edu.pl; 2 Division of Clinical Anatomy, Medical University of Gdańsk, Dębinki 1, 80-211 Gdańsk, Poland; adam.kosinski@gumed.edu.pl

**Keywords:** right ventricular outflow tract, RVOT, digital photogrammetry, 3D point-cloud models, quantitative geometry analysis, cross-sectional analysis, anatomical modelling, cardiac morphology

## Abstract

The right ventricular outflow tract (RVOT) is an anatomically complex and spatially variable cardiac region that is difficult to characterise using isolated manual measurements. Although three-dimensional reconstruction techniques enable detailed visualisation of RVOT morphology, reproducible workflows for quantitative, level-based analysis of RVOT geometry remain limited. The aim of this methodological study was to develop and evaluate a semi-automated workflow for quantitative analysis of RVOT geometry based on 3D point-cloud models. This study represents a secondary methodological analysis of an existing anatomical dataset, introducing a new semi-automated workflow for systematic cross-section generation and quantitative descriptor extraction. Anatomical RVOT models were prepared from post-mortem material using silicone casting and digital photogrammetry. After point-cloud preparation, primary cross-sections were used to estimate a global directional reference. A mathematically defined inferior boundary plane and a predefined superior anatomical limit were then used to generate secondary cross-sections along the standardised RVOT segment. For each retained section, positional, ellipse-based, distance-based and area-based descriptors were extracted. The workflow was applied to 60 RVOT point-cloud models. After quality control, 661 secondary cross-sections were retained for analysis, with 5–16 sections per model and a mean of 11.02 ± 2.47 sections per model. Methodological validation included synthetic testing, comparison with manual measurements, and inter-observer assessment. Agreement with manual dimensions was high (ICC = 0.905–0.937), while inter-observer landmark variability was low (RMSE = 0.41 mm). An additional exploratory inter-observer assessment of three RVOT models yielded mean model-specific RMSE values of 12.97 mm^2^ for cross-sectional area and 0.54 mm for both ellipse axes. The proposed workflow provides a predefined geometric framework for quantitative description of RVOT geometry and should be regarded as a methodological proof of concept rather than a clinically validated measurement system.

## 1. Introduction

The right ventricular outflow tract (RVOT) is a cardiac structure of major importance in interventional cardiology and cardiac surgery, serving, among other roles, as a key target for the catheter ablation of idiopathic ventricular arrhythmias and as an optimal site for implantation of cardiac pacing leads [1,2]. Despite its important clinical role, the RVOT remains a structure that is extremely difficult to describe in precise geometric terms due to its irregular, asymmetric shape, curved three-dimensional course, and complex internal architecture, which comprises a smooth-walled conus arteriosus and a trabeculated proximal portion [3,4].

Most morphometric studies of the RVOT have relied on simplified geometric descriptions based on selected linear dimensions or manually defined cross-sectional measurements rather than comprehensive three-dimensional shape analysis [5,6]. Although this approach is useful in routine imaging diagnostics (e.g., two-dimensional echocardiography), it does not allow for a complete representation of the true three-dimensional geometry of this structure. Traditional manual measurements are largely subjective and prone to inter-observer variability, which hampers data standardisation. Moreover, conventional morphometric analysis rarely accounts for the fact that the RVOT is characterised by a curved course and a significant rotational configuration. As a result, standard cross-sectional planes may not be orthogonal to its central axis, potentially leading to measurement distortion [7].

Previous studies have shown that three-dimensional reconstruction methods can be used to visualise the spatial structure of the right ventricular cavity and the RVOT [6]. In particular, silicone casting combined with digital photogrammetry has enabled the creation of anatomically detailed 3D models of the RVOT, allowing its complex morphology to be assessed beyond conventional two-dimensional observation. These approaches have provided important insight into the spatial configuration and variability of the RVOT; however, quantitative analyses have generally focused on selected geometric characteristics rather than on systematic, multidimensional characterisation of successive cross-sections along a standardised RVOT segment.

Despite these advances, there remains a methodological gap between three-dimensional visualisation of RVOT anatomy and structured quantitative analysis of RVOT geometry. Although 3D reconstruction provides anatomically detailed models, such models do not automatically yield comparable quantitative information. The RVOT is not a regular cylindrical structure, and its cross-sectional size, shape and orientation may change along the length of the outflow tract. Therefore, isolated linear measurements or manually selected single-level assessments may not adequately describe its spatial geometry. A reproducible workflow is needed to standardise RVOT 3D point-cloud models and convert them into a level-based dataset of cross-sections and geometric descriptors that describe local size, shape, outline deviation and asymmetry.

A previous study by our group investigated the three-dimensional configuration of the RVOT using the same 60 adult human hearts and the same 3D point-cloud models analysed in the present study [8]. That study focused specifically on the spatial distribution of cross-sectional centroids, their displacement relative to the RVOT axis, and differences in centroid position between predefined age groups. The present study builds on this previously described anatomical material as the basis for a distinct methodological investigation. Rather than analysing centroid displacement as the primary outcome, the present workflow is designed to standardise the RVOT models and generate a series of secondary cross-sections along a model-specific reference axis, followed by systematic extraction of multiple geometric descriptors describing cross-sectional position, size, shape, outline deviation and local asymmetry. Thus, the previously published age-group analysis and centroid-based findings are not reproduced as the primary results of the present study. The methodological contribution of the present work lies in the transformation of three-dimensional RVOT point-cloud geometry into a structured, level-based descriptor dataset that can be used for subsequent quantitative analyses.

Although the same anatomical material and 3D point-cloud models were used, the present study addresses a distinct methodological objective. In the previous study [8], cross-sections were manually selected and measured, whereas the present study introduces a semi-automated workflow for systematic generation of consecutive secondary cross-sections and computational extraction of multiple geometric descriptors along the analysed RVOT segment (Table 1). Accordingly, the novelty of the present study lies in the analytical workflow and its methodological validation rather than in the anatomical dataset itself. The figures and validation analyses presented in the current manuscript were prepared specifically for this study, while the basic characteristics of the anatomical dataset remain unchanged.

The methodological contribution of the present study should be distinguished from existing approaches to three-dimensional anatomical reconstruction, statistical shape modelling, and conventional cross-sectional analysis. While 3D reconstruction enables detailed visualisation of RVOT morphology, it does not inherently provide a structured quantitative description of geometric variation along the outflow tract. Statistical shape modelling approaches primarily aim to characterise population-level an-atomical variability, whereas conventional section-based analyses often focus on selected anatomical levels or individual geometric measurements.

The present study does not introduce a new 3D reconstruction technique or a novel mathematical algorithm for geometric fitting. Instead, its methodological contribution lies in the integration of established computational techniques into a semi-automated workflow adapted to RVOT anatomy. Specifically, the workflow combines SVD-based estimation of a model-specific directional reference, anatomically guided definition of the inferior RVOT boundary, generation of secondary cross-sections perpendicular to the standardised axis, and systematic extraction of positional, ellipse-based, distance-based, and area-based descriptors.

This integrated approach enables the transformation of complex RVOT point-cloud geometry into a structured, level-based quantitative dataset, allowing geometric characteristics to be assessed across consecutive sections of the analysed RVOT segment. The proposed workflow therefore provides a methodological framework for systematic cross-sectional characterisation of RVOT geometry rather than a replacement for existing reconstruction or statistical shape modelling methods.

## 2. Materials and Methods

### 2.1. Study Material and Ethical Approval

The study was conducted using 60 adult human hearts of both sexes (15 females and 45 males), aged 18 to 85 years (mean age: 44.6 ± 18.2 years). The specimens originated from individuals who had participated in a voluntary body donation programme for scientific purposes. Following donation, the hearts were transferred to the anatomical collection of the Division of Clinical Anatomy at the Medical University of Gdańsk, Poland. Specimen collection covered a four-year period.

The hearts were selected consecutively from the available anatomical specimens, provided that they met the predefined inclusion criteria. These included a non-cardiovascular cause of death, a negative medical history of cardiovascular disease, and the absence of macroscopically identifiable pathological changes during anatomical examination. The available specimen records included age, sex, date of death, heart weight, and information regarding the non-cardiovascular cause of death. Detailed medical records, individual autopsy procedures, post-mortem intervals, and fixation durations were not available to the research team. Although detailed information regarding the fixation procedure and its duration was not available to the research team, according to the institutional procedures in place at the time of specimen collection, the interval between donor death and the initiation of specimen fixation did not exceed 48 h.

The hearts were fixed in a formalin-based solution prepared using 10% formalin and 98% (*v*/*v*) ethanol. Following fixation, the specimens were stored in appropriately sized plastic containers to avoid mechanical compression and deformation of the cardiac structures.

### 2.2. Silicone Casting and Photogrammetric 3D Reconstruction

The preparation of silicone casts and the photogrammetric reconstruction protocol have been described in detail in previous studies [9,10]. The photogrammetric reconstruction approach used in the present study was based on a previously described workflow for silicone models of the cardiac cavities, in which digital photogrammetry was used to obtain scaled 3D models suitable for anatomical measurement and analysis [10].

Briefly, silicone casts of the right ventricular cavity, including the RVOT, were prepared from the anatomical specimens using a previously established casting procedure. Before casting, the cardiac chambers were rinsed with water to remove residual blood clots. The venous openings of the right atrium were sutured, and a balloon catheter was positioned within the pulmonary trunk, with the balloon located above the pulmonary valve to minimise deformation of the RVOT and pulmonary trunk during casting. The right ventricular cavity was filled with a two-component silicone moulding compound (XIAMETER™ 4250-S Green silicone, The Dow Chemical Company, Midland, MI, USA).) using a gravity-assisted filling technique.

During the casting procedure, each heart was positioned in a container with the ventricular apex directed upwards. The silicone mixture was introduced slowly through an incision at the ventricular apex using a 100 mL Janet syringe (ZARYS International Group, Zabrze, Poland), allowing gravity-assisted filling of the cardiac chambers without the application of controlled injection pressure. This approach was intended to minimise mechanical distension of the cardiac walls and facilitate the penetration of silicone into the internal anatomical structures.

During filling, gentle compression of the cardiac walls was applied to facilitate the removal of air bubbles and improve the reproduction of the internal anatomical structures. The silicone was allowed to polymerise for 24 h at room temperature. Following polymerisation, the silicone casts were carefully extracted by incising the walls of the right ventricle and right atrium. Following removal from the cardiac specimens, the casts were left for an additional 24 h before further processing.

The resulting silicone casts were photographed from multiple viewpoints and reconstructed as 3D surface models using a digital photogrammetry workflow [6]. Image acquisition was performed in 2019 using a Sony Alpha DSLR-A300 digital camera (Sony Corporation, Tokyo, Japan) equipped with a Sony 18–70 mm f/3.5–5.6 lens. For each silicone cast, a series of 50 photographs was acquired using a custom-designed rotating platform. The cast was incrementally rotated between successive photographs to capture its external surface from multiple viewing angles. The acquired photographs were imported into Autodesk ReCap Photo v19.2.0.16 (Autodesk, Inc., San Francisco, CA, USA) for photogrammetric reconstruction and generation of three-dimensional polygonal surface meshes.

The resulting models were scaled, visually inspected and cleaned to remove reconstruction artefacts. Scale calibration was performed using reference distances measured directly between selected landmarks on the corresponding physical silicone cast. The same landmarks were identified on the digital model, and the measured distances were used to establish the appropriate spatial scale. Reconstruction artefacts, including unwanted fragments of the rotating platform, were manually removed using Autodesk ReCap Photo. The resulting polygonal meshes were subsequently converted into point-cloud representations for further geometric processing.

This workflow provided spatially accurate 3D representations of the right ventricular cavity and RVOT, which were subsequently used for point-cloud preparation and semi-automated geometric analysis.

### 2.3. Point-Cloud Preparation in CloudCompare

The reconstructed 3D surface models were imported into CloudCompare v2.11.3 for visual inspection and preparation of the RVOT point-cloud data. Reconstruction artefacts and non-relevant fragments of the model were removed manually. The RVOT region was then isolated from the cast of the right ventricle, while preserving the anatomical region required for subsequent definition of the inferior RVOT boundary.

The cleaned 3D models were converted into point-cloud datasets and checked for scale consistency and completeness. The point-cloud datasets were generated from the OBJ surface-mesh models and were characterised by an approximately uniform spatial point distribution over the model surface, rather than by strongly non-uniform sampling density. The superior border of the RVOT was identified at the level of the ring connecting the attachment points of the pulmonary valve leaflets. The anatomical region associated with the supraventricular crest was also preserved, as it was required for subsequent placement of the inferior boundary plane.

**Figure 1:**
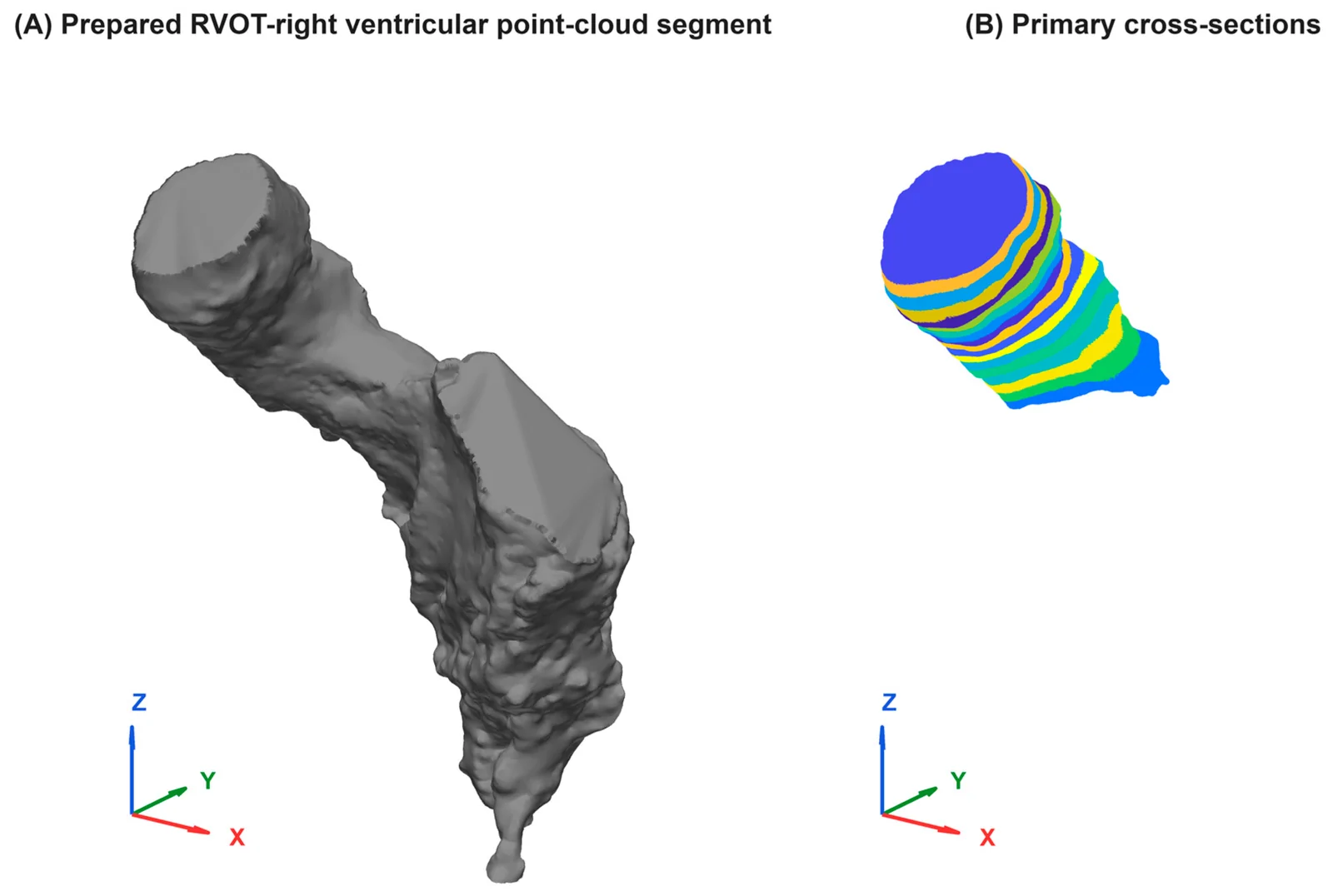
Point-cloud preparation for RVOT analysis in CloudCompare. (**A**) Shaded point-cloud representation of the prepared right ventricular cast segment, including the RVOT and the adjacent ventricular region required for anatomical identification of the inferior RVOT boundary. (**B**) Primary cross-sections generated parallel to the superior anatomical border of the RVOT.

No final geometric descriptors were calculated at this stage. CloudCompare was used to prepare scaled and cleaned RVOT point clouds and to generate the primary cross-sections used as input (Figure 1) for the MATLAB-based semi-automated workflow described in Section 2.4.

Manual preparation in CloudCompare was retained at this stage because the RVOT casts showed marked inter-individual variability and irregular surface geometry, making fully automated isolation of anatomically consistent RVOT regions unreliable.

### 2.4. Semi-Automated Workflow for RVOT Point-Cloud Analysis

The semi-automated workflow combined anatomical landmark identification with axis-based geometric standardisation of RVOT point clouds. Manual steps included point-cloud preparation, preparation of primary cross-sections, anatomical landmark identification and visual quality control of the generated sections. Centroid calculation, directional line estimation, secondary section generation and descriptor extraction were performed computationally.

#### 2.4.1. Input Data for the RVOT Point-Cloud Analysis

For each RVOT, the input dataset consisted of a 3D point cloud calibrated to the physical dimensions of the corresponding silicone cast, with coordinates expressed in millimetres of the isolated outflow tract, a set of primary cross-sections prepared in CloudCompare, and an anatomical reference point related to the supraventricular crest. The point cloud represented the surface geometry of the RVOT obtained from the photogrammetry-based reconstruction of the silicone cast.

The primary cross-sections were generated parallel to the plane of the superior RVOT boundary, defined at the level of the ring connecting the attachment points of the pulmonary valve leaflets. These sections were not used as final analytical sections but as an initial geometric sampling of the RVOT. Their centroids were subsequently used to estimate the directional line of the structure.

The anatomical reference point corresponded to the characteristic indentation associated with the supraventricular crest. It was used to anchor the inferior boundary of the RVOT during the subsequent axis-based standardisation of the model.

#### 2.4.2. Primary Cross-Sections and Centroid Extraction

Primary cross-sections were generated in CloudCompare v2.11.3 as consecutive 2-mm-thick slices of the RVOT point cloud. The position of the first section was defined manually, after which subsequent sections were generated successively using the CloudCompare sectioning tool. The section advance was 2 mm; therefore, adjacent sections were contiguous, with coincident boundaries and no overlap or gaps. All primary sections were oriented parallel to the plane of the superior RVOT boundary, defined at the level of the pulmonary valve leaflet attachment ring. This step provided an initial and consistent sampling of the RVOT geometry before axis-based standardisation.

Each primary cross-section was exported as a separate set of 3D points and processed in MATLAB R2023a. For every primary cross-section, the centroid was calculated as the arithmetic mean of the coordinates of all points belonging to that section:

(1) $$c_{i}=\frac{1}{n_{i}}\overset{n_{i}}{\underset{j=1}{\sum }}p_{ij}$$

where $c_{i}$ is the centroid of the *i*-th primary cross-section, *n_i_* is the number of points in that section, and $p_{ij}=\left(x_{ij},y_{ij},z_{ij}\right)$ denotes the *j*-th point of the *i*-th section.

The resulting sequence of centroids represented the spatial displacement of consecutive RVOT levels and was used in the next step to estimate the directional line of the structure.

#### 2.4.3. Directional Line Estimation

**Figure 2:**
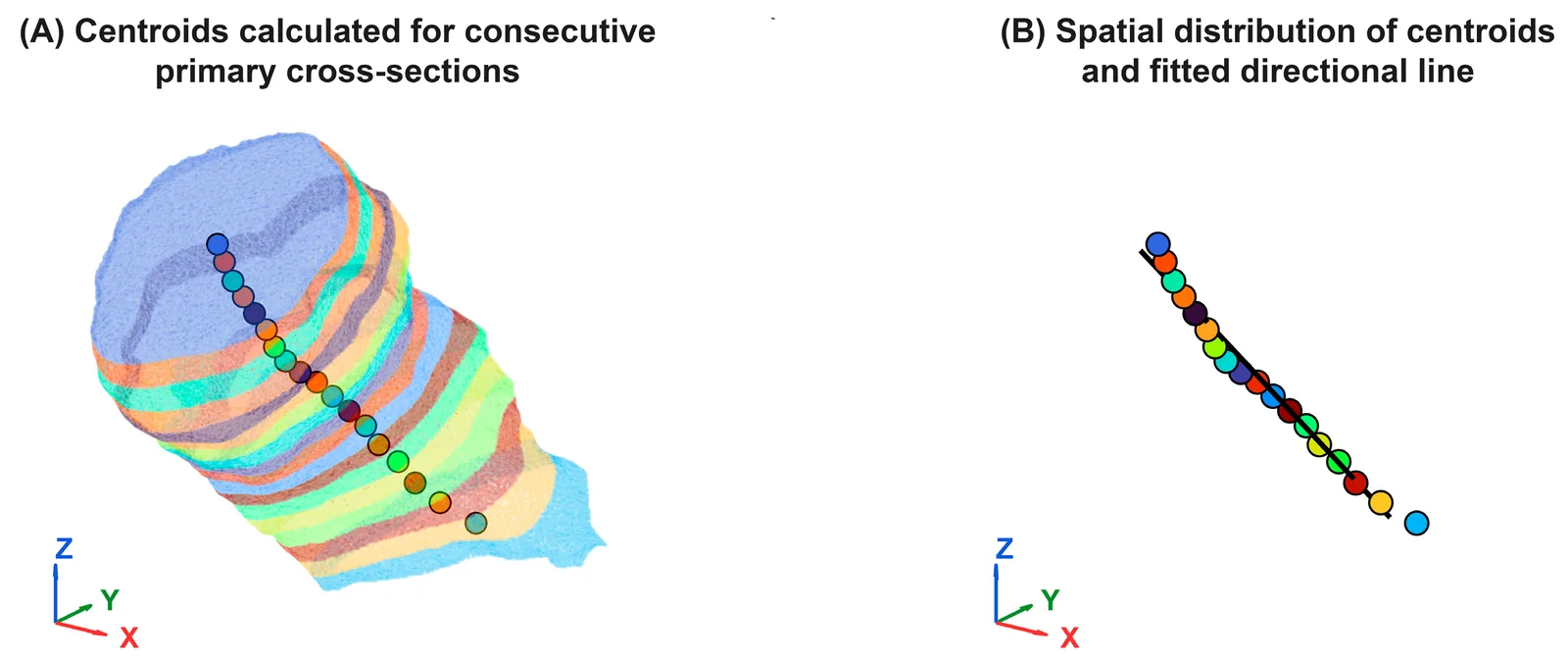
Primary-section centroids and directional line estimation. (**A**) Primary 2-mm RVOT cross-sections generated parallel to the superior boundary plane, shown as semi-transparent point sets, with the centroid of each cross-section marked by a larger point in the corresponding colour. (**B**) Spatial distribution of the primary-section centroids used for directional line estimation. The black line represents the SVD-based orthogonal least-squares fitted directional line.

The centroids of the primary cross-sections were used to estimate the main spatial orientation of the RVOT. As the centroid coordinates did not form a collinear set, a best-fit line was calculated using an SVD-based orthogonal least-squares approach, consistent with established methods for fitting geometric elements to three-dimensional point data [11]. Briefly, the centroid coordinates were centred by subtracting their mean position, and singular value decomposition was applied to the centred coordinate matrix. The first right singular vector was used as the direction vector of the fitted line. The fitted line was defined as a model-specific global directional reference. It was not intended to represent the local anatomical centreline of the RVOT, but to provide a consistent longitudinal axis for model standardisation and generation of mutually parallel secondary cross-sections:

(2) $$l\left(t\right)=\bar{c}+tv$$

where $l\left(t\right)$ represents a point on the fitted line, $\bar{c}$ is the mean position of all primary-section centroids, v is the direction vector of the fitted line, and t is a scalar parameter. The directional line was used as a model-specific geometric reference for boundary plane definition and generation of secondary cross-sections. The primary-section centroids and the resulting SVD-based fitted directional line are shown in Figure 2.

#### 2.4.4. Inferior Boundary Plane Definition and Model Standardisation

**Figure 3:**
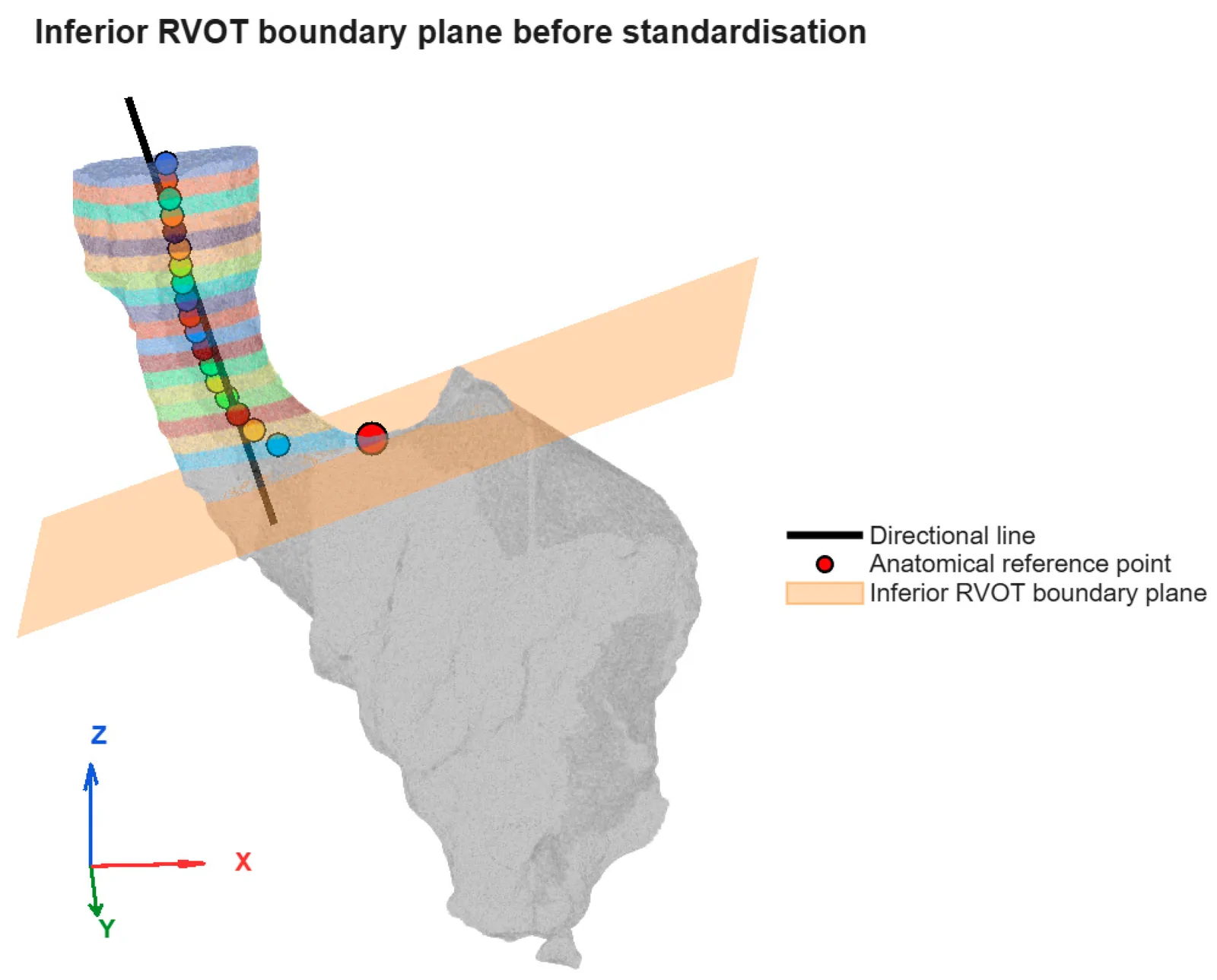
Definition of the inferior RVOT boundary plane before model standardisation. Prepared point-cloud model of the right ventricular cast showing the primary RVOT cross-sections and their centroids used for directional line estimation. The black line represents the SVD-based fitted directional line. The red point marks the anatomical reference point associated with the supraventricular crest, through which the inferior boundary plane was defined. The orange plane represents the mathematically defined inferior RVOT boundary, perpendicular to the directional line.

After estimating the directional line, the RVOT model was standardised with respect to its model-specific axis. The direction vector of the fitted line was used as the normal vector for defining the inferior boundary plane. The inferior RVOT boundary was defined as a plane passing through the anatomical reference point associated with the supraventricular crest and perpendicular to the directional line:

(3) $$\Pi_{L}:v\cdot \left(p-p_{\mathrm{SC}}\right)=0$$

where $\Pi_{L}$ is the inferior boundary plane, v is the direction vector of the fitted line, p is any point on the plane, and $p_{\mathrm{SC}}$ is the anatomical reference point associated with the supraventricular crest. The spatial relationship between the fitted directional line, the anatomical reference point and the resulting inferior RVOT boundary plane is shown in Figure 3.

The region extending from the inferior boundary plane to the predefined superior anatomical limit was then used for the generation of secondary cross-sections.

#### 2.4.5. Generation of Secondary Cross-Sections

After estimation of the model-specific directional line and definition of the inferior RVOT boundary plane, the point-cloud model was transformed into a standardised coordinate system. In this transformation, the directional line was used as the reference axis of the RVOT, and the inferior boundary plane, defined through the anatomical reference point associated with the supraventricular crest, was transformed together with the model. This step enabled secondary cross-sections to be generated in a consistent orientation perpendicular to the standardised RVOT axis.

Secondary cross-sections were then generated between the mathematically defined inferior boundary plane and the predefined superior anatomical limit of the RVOT. Section generation started at the inferior RVOT boundary plane and proceeded along the model-specific directional axis towards the superior anatomical limit. These sections were defined as the final analytical cross-sections used for quantitative geometric assessment.

Each secondary cross-section was created as a 3-mm-thick slice of the RVOT point cloud. The section advance was 3 mm; therefore, consecutive sections were contiguous, with coincident boundaries and no overlap or gaps. This thickness was selected to preserve local geometric information while retaining a sufficient number of points for stable outline reconstruction and area calculation. The 3-mm thickness represents the parameter used in the present study rather than a fixed requirement of the workflow and may be adjusted according to point density, object scale and the intended application. The sections were generated at successive positions along the standardised directional axis, with each section including all point-cloud points located within the corresponding 3-mm interval.

**Figure 4:**
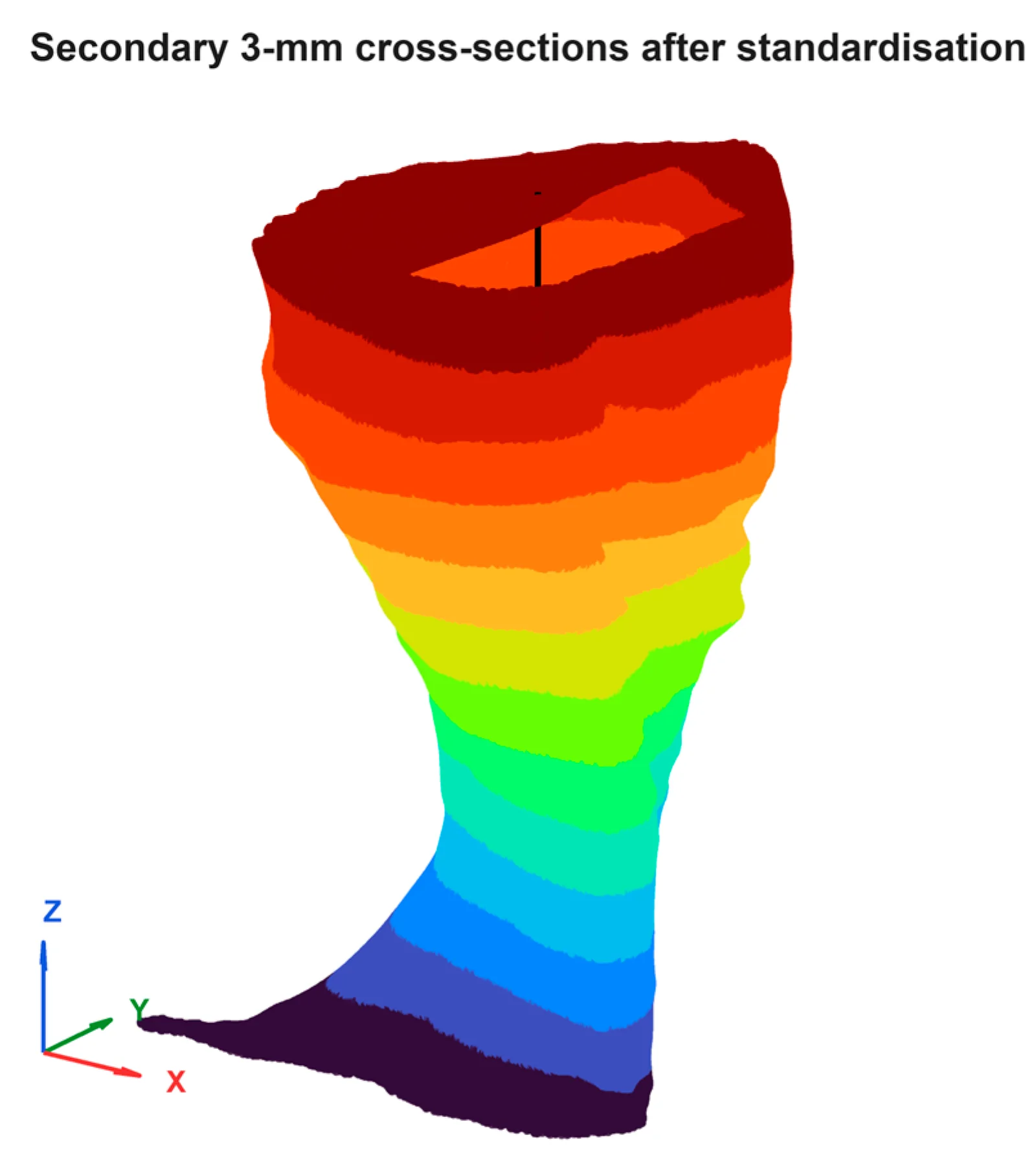
Secondary RVOT cross-sections generated after model standardisation. Series of 3-mm secondary cross-sections generated along the transformed directional axis between the mathematically defined inferior RVOT boundary and the predefined superior anatomical limit. The sections represent the final analytical cross-sections used for quantitative geometric assessment after visual quality control.

As a quality-control step, the generated secondary cross-sections were visually inspected by the operator. Sections were excluded from further analysis when they did not represent a complete local RVOT cross-section, most commonly at the terminal part of the analysed segment, where the local course and inclination of the RVOT resulted in only a partial wall representation. The number of retained secondary cross-sections therefore varied between models, depending on the length and geometry of the analysed RVOT segment. To reconstruct the section-generation log, the generation step was subsequently re-run for all 60 RVOT models using the original model-specific input data and sectioning parameters. The number of generated sections was recorded for each model and compared with the number of sections retained in the final analytical dataset. The number of excluded sections was calculated as the difference between these values. The resulting series of secondary 3-mm cross-sections generated after model standardisation is shown in Figure 4.

#### 2.4.6. Extraction of Geometric Descriptors

Each secondary cross-section was analysed as a two-dimensional point set after projection onto its section plane. The extracted descriptors were organised into four groups: positional descriptors, ellipse-based descriptors, distance-based descriptors and area-based descriptors. This structure was used to describe each section consistently and to enable comparison between consecutive RVOT levels.

For each section, the centroid was calculated as the arithmetic mean of the projected point coordinates. Because the analysed point clouds originated from approximately uniformly sampled surface-mesh models, this definition was expected to be less susceptible to local sampling-density bias than in irregularly sampled point clouds. Its sensitivity to centroid definition and contour resampling was nevertheless evaluated separately as part of the methodological validation. Least-squares ellipse fitting is a well-established approach for geometric approximation of planar point sets [12,13]. In the present study, ellipse fitting was performed using the fit_ellipse function by Gal [14], which applies an unweighted least-squares fit to the conic representation of an ellipse. The fit was considered unsuccessful when the fitted conic did not represent an ellipse and valid ellipse parameters were therefore not returned.

The cross-sectional boundary was reconstructed from the two-dimensional point set using the MATLAB R2023a boundary function with the default shrink factor of 0.5, and the enclosed area was calculated using polyarea. No additional automatic outlier-removal procedure was applied before boundary reconstruction or ellipse fitting.

The long and short dimensions were defined as twice the larger and smaller fitted semi-axes, respectively. The ellipse orientation parameter *θ*, describes the orientation of the fitted ellipse within the local cross-sectional coordinate system. Because ellipse axes are undirected, the orientation has an inherent 180° ambiguity. The fitted ellipse was used as a standardised geometric descriptor and was not intended to imply that the anatomical cross-section was intrinsically elliptical. Distance-based descriptors were used to describe local deviations of the section outline from its centre and from the fitted ellipse centre. Area-based descriptors included the measured section area, the fitted ellipse area, the difference between these areas and the areas of four quadrants defined by the axes of the fitted ellipse.

The full set of descriptors extracted from each secondary cross-section is summarised in Table 2. Quadrants I–IV were defined exclusively within the local coordinate system of the fitted ellipse and do not correspond to fixed anatomical directions. Therefore, individual quadrant areas should not be interpreted as anatomically corresponding regions across different RVOT models.

**Figure 5:**
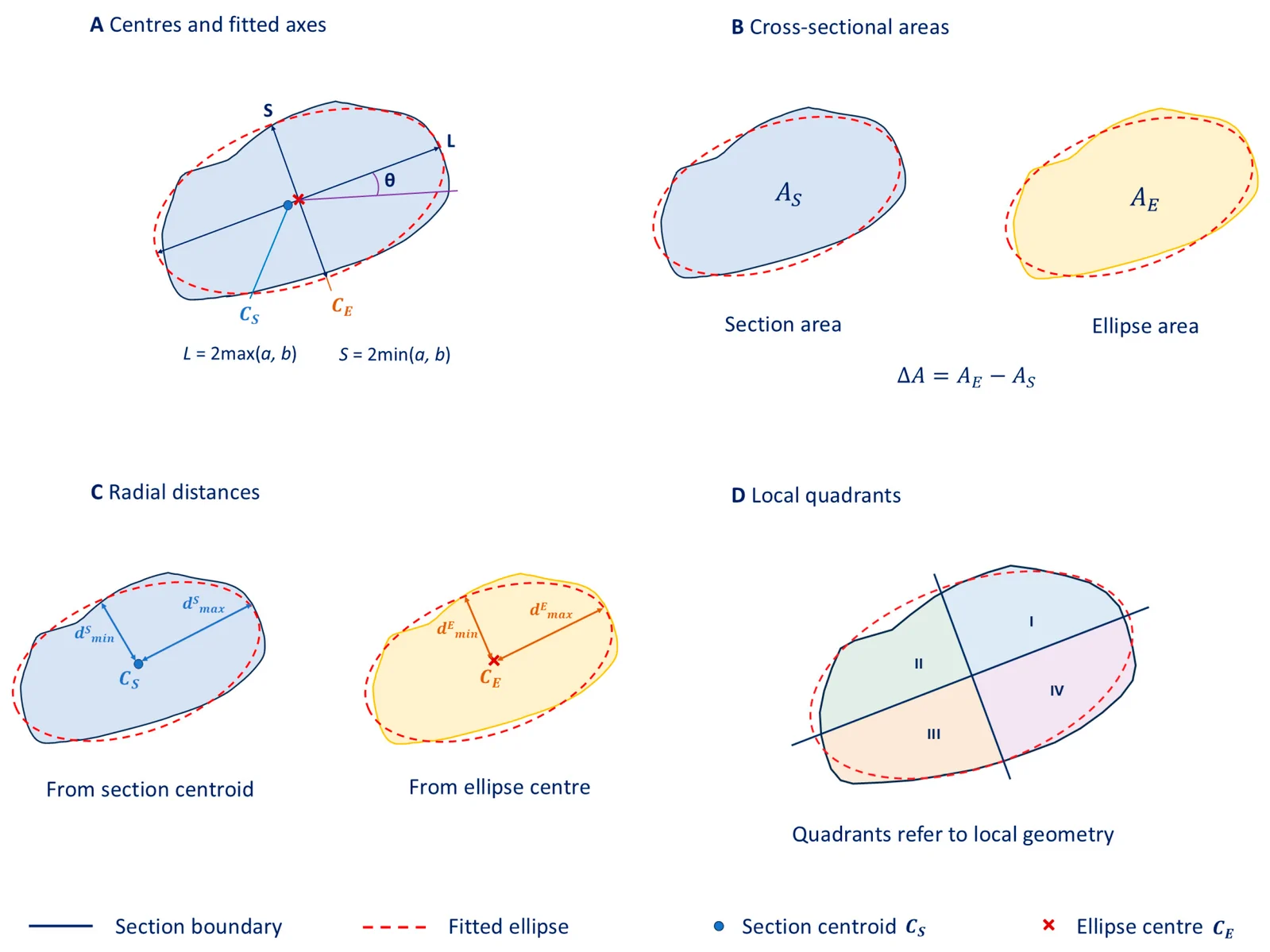
Schematic illustration of the geometric descriptors extracted from secondary RVOT cross-sections.

All descriptors were exported as structured numerical tables, with each row corresponding to one secondary cross-section and each column corresponding to one geometric descriptor. These tables constituted the input dataset for subsequent statistical analysis. The principal geometric descriptors listed in Table 2 are illustrated schematically in Figure 5.

### 2.5. Methodological Validation Procedures

The workflow was evaluated using four complementary validation procedures. First, numerical correctness and robustness of the cross-sectional descriptors were assessed using ideal and perturbed synthetic cross-sections. The Monte Carlo robustness analysis comprised 462 synthetic cross-sections generated from two representative elliptical geometries under Gaussian coordinate noise, outlier contamination, incomplete contours and variation in point density; a separate deterministic analysis assessed geometric variation within the 3-mm section thickness. Additional sensitivity analyses using the same synthetic dataset assessed the effects of boundary shrink factor, centroid definition and contour resampling. Boundary-derived area was recalculated for shrink factors of 0.25, 0.50 and 0.75, while the arithmetic-mean centroid was compared with the polygon area centroid and with the centroid obtained after uniform arc-length resampling to 120 contour points. The effect of resampling on boundary-derived area was also quantified. Second, the suitability of the global SVD-based reference axis was assessed in a subset of 15 of the 60 RVOT models, selected without applying any model-specific selection criterion and without prior inspection of model geometry or validation results, comprising 224 primary-section centroids. Third, ellipse-derived long and short dimensions were compared with independent manual measurements in 45 secondary cross-sections obtained from the same 15 models, with one section selected from each of the inferior, middle and superior portions of each model without applying morphology-based selection criteria or inspecting the automated measurement results. Fourth, inter-observer variability in anatomical landmark identification was assessed in the same 15 RVOT models using independent selections performed by two operators. Agreement was evaluated using difference-based statistics, Bland–Altman limits of agreement and ICC(A,1), as appropriate. Detailed validation procedures and complete results are provided in Supplementary Materials S1.

An additional exploratory inter-observer assessment was performed using three RVOT point-cloud models, designated RVOT-A, RVOT-B, and RVOT-C. Two observers processed each model using the semi-automated workflow. The resulting datasets were compared with respect to cross-sectional area, ellipse major and minor axes, ellipse area, and the difference between cross-sectional and ellipse areas. Retained cross-sections were paired according to their sequential positions within each model. The section index was normalised to the interval [0, 1], corresponding to the first and last retained sections, respectively. Agreement was quantified using the signed mean difference (bias; Observer 2 minus Observer 1), mean absolute error (MAE), and root mean square error (RMSE). These statistics were calculated separately for each RVOT model and subsequently averaged with equal weighting of the three models. Given the limited number of independent anatomical specimens and the nested structure of the section-level measurements, this assessment was considered exploratory. The numbers of generated, excluded, and retained secondary cross-sections were recorded separately for each observer. Total processing time, encompassing both manual and computational operations, was also recorded for each processing run.

### 2.6. Statistical Analysis

The statistical analysis was designed to summarise the quantitative output of the semi-automated workflow and to demonstrate its ability to describe geometric variation along the standardised RVOT segment. The analysis was descriptive and methodological in nature and was not intended to perform clinical subgroup comparisons.

The number of retained secondary cross-sections was calculated for each RVOT model, together with the total number of descriptor records generated by the workflow. The number of sections per model was summarised using mean, standard deviation, median, minimum and maximum values.

To enable comparison of corresponding positions between RVOT models containing different numbers of retained secondary cross-sections, section position was normalised within each model to a relative RVOT level ranging from 0 to 1. For model i, containing $n_{i}$ retained secondary sections ordered from inferior to superior, the relative level of section k was calculated as:

$$r_{i,k}=\frac{k-1}{n_{i}-1},\;\;k=1,\ldots ,n_{i}$$

where $r_{i,k}$ is the relative RVOT level. Consequently, the first retained secondary section was assigned a value of 0 and the last retained section a value of 1, with the remaining sections positioned proportionally according to their sequential order. The normalisation was therefore based on section order within each model rather than on absolute RVOT length.

Three representative sections were additionally selected from each model for descriptive comparison. The inferior level was defined as the first retained secondary section (k=1), the superior level as the last retained section $\left(k=n_{i}\right),$ and the mid-level as the section with index:

$$k_{\mathrm{mid}}=\mathrm{round}\left(\frac{n_{i}+1}{2}\right).$$

For models containing an even number of sections, this rule selected the superior of the two central sections. Each of the 60 RVOT models therefore contributed one observation to each of the inferior, mid-level and superior summaries.

For representative geometric descriptors, descriptive statistics were calculated at the inferior, mid-level and superior levels of the standardised RVOT segment. These levels were used to demonstrate level-based analysis of RVOT geometry. Cross-sectional area was selected as the main representative descriptor for visualisation because it provides an intuitive measure of local RVOT size. Additional ellipse-based and distance-based descriptors were summarised to demonstrate that the workflow generated a broader multi-parameter geometric description.

To account for repeated sections within individual hearts, cross-sectional area was linearly interpolated within each RVOT model to common relative levels from 0 to 1 at intervals of 0.1. Median and interquartile range values were then calculated across the 60 heart-level values at each relative level, giving each RVOT model equal weight.

Because the 45 validation sections were nested within 15 hearts, 95% confidence intervals for ICC(A,1) were estimated using heart-clustered bootstrap resampling (10,000 resamples), with all three sections from each resampled heart retained together.

All statistical analyses and graphical summaries were performed in MATLAB R2026a. No hypothesis testing of anatomical or clinical subgroup differences was performed.

### 2.7. Use of Generative Artificial Intelligence

Generative artificial intelligence tools were used for superficial language editing and verification of English wording and translation consistency.

## 3. Results

### 3.1. Application of the Semi-Automated Workflow to the RVOT Dataset

The semi-automated workflow was applied to 60 RVOT point-cloud models. Rerunning the section-generation step produced 687 secondary cross-sections in total. Following quality control, 26 sections (3.8%) were excluded and 661 sections were retained for quantitative analysis. The number of retained secondary cross-sections ranged from 5 to 16 per model, with a mean of 11.02 ± 2.47 and a median of 11 sections. Model-specific numbers of generated, excluded and retained sections are provided in Supplementary Materials, Table S7. This output confirmed that the proposed workflow could be applied to the full RVOT model dataset and that it generated a structured set of comparable cross-sectional records for subsequent geometric description. The quantitative output of the workflow is summarised in Table 3.

### 3.2. Descriptor Dataset Generated from Secondary Cross-Sections

Each retained secondary cross-section was represented by a structured set of geometric descriptors, including positional, ellipse-based, distance-based and area-based parameters. The final dataset consisted of 661 descriptor records, with each record corresponding to one secondary RVOT cross-section. This dataset provided the basis for a descriptive demonstration of geometric variation along the standardised RVOT segment.

Cross-sectional area was selected as a representative descriptor for visualisation because it provides an intuitive measure of local RVOT size and can be directly compared along the relative RVOT level. The purpose of this analysis was not to perform clinical subgroup comparisons, but to demonstrate that the workflow enables quantitative tracking of geometric descriptors along a standardised RVOT segment. The same level-based approach can be applied to the remaining ellipse-based, distance-based and asymmetry descriptors generated by the workflow.

### 3.3. Representative Visualisation of Cross-Sectional RVOT Geometry

**Figure 6:**
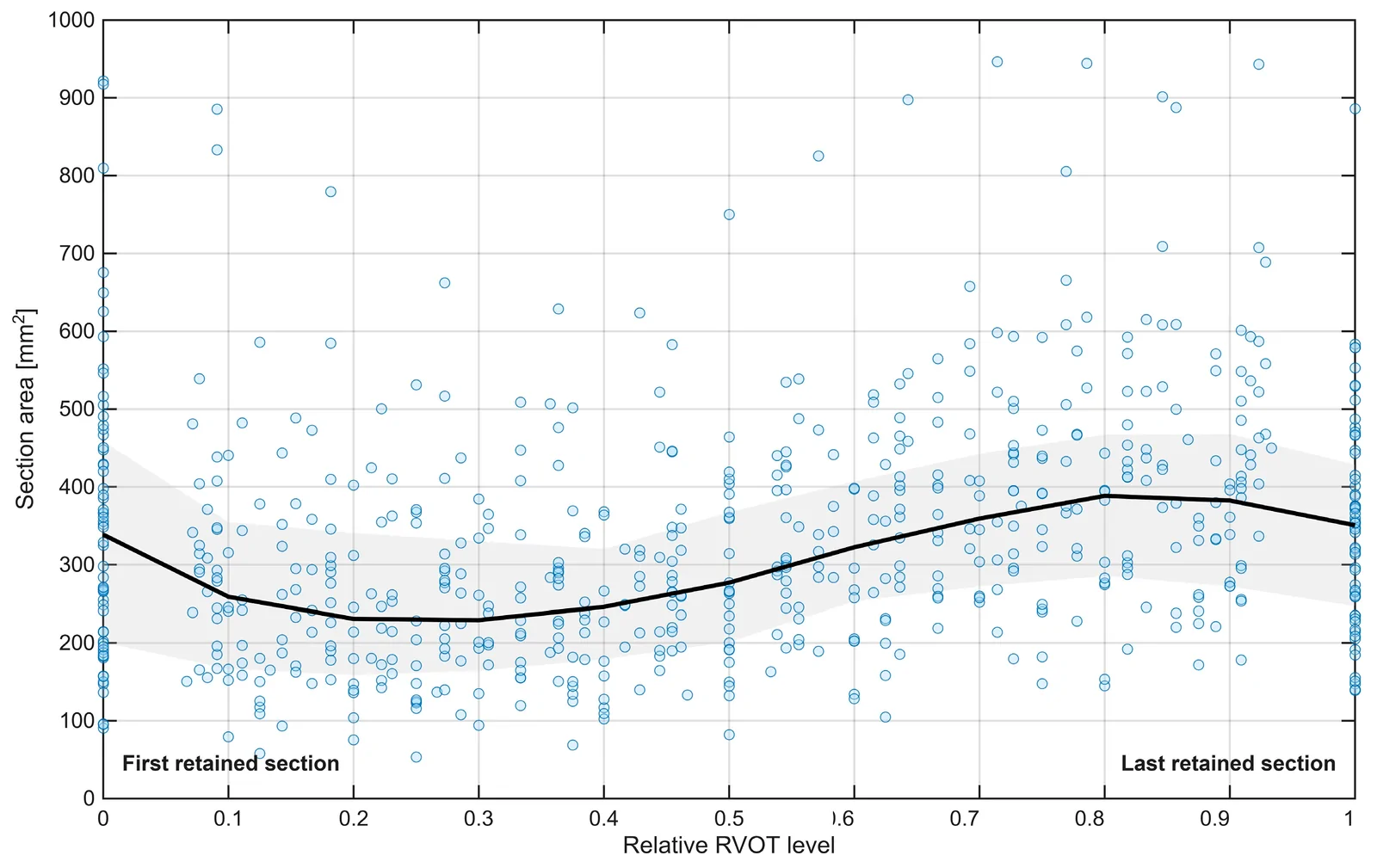
Distribution of RVOT cross-sectional area along the relative level of the analysed segment. Each point represents one secondary cross-section retained after quality control. The relative RVOT level ranges from 0, corresponding to the first retained secondary section, to 1, corresponding to the last retained secondary section of each model. The black line represents the median section area calculated across heart-level values at common relative levels, and the shaded area represents the corresponding interquartile range (*n* = 60 RVOT models at each level).

The distribution of cross-sectional area values along the relative RVOT level is shown in Figure 6. Each point represents one secondary cross-section retained after quality control. The relative RVOT level ranges from 0, corresponding to the first retained secondary section, to 1, corresponding to the last retained secondary section of each model.

To account for the clustering of repeated sections within individual hearts, section area was interpolated within each RVOT model to common relative levels from 0 to 1 at intervals of 0.1. The median and interquartile range were then calculated across the 60 heart-level values at each relative level. The original section-level observations are shown as individual points, whereas the summary profile gives each RVOT model equal weight.

The plot demonstrates that cross-sectional area can be compared across models despite inter-individual differences in the absolute length of the RVOT segment. Section area values showed marked variability along the analysed segment, supporting the need for a standardised, level-based approach to quantitative RVOT description rather than relying on isolated manual measurements.

### 3.4. Descriptive Assessment of Selected Representative Descriptors

Selected descriptors were summarised at three representative levels of the RVOT: inferior, mid-level and superior. These levels were used to demonstrate the analytical output of the workflow and were not intended as clinical subgroup comparisons. The results are presented in Table 4.

The mean cross-sectional area was 356.55 ± 190.40 mm^2^ at the inferior level, 302.72 ± 121.71 mm^2^ at the mid-level and 350.50 ± 138.84 mm^2^ at the superior level. The corresponding median values were 338.61 mm^2^, 289.47 mm^2^ and 350.69 mm^2^, respectively. These values demonstrate that the workflow can quantify local RVOT size at defined relative levels of the analysed segment.

Ellipse-based descriptors showed that the workflow also captured shape-related information. The long axis was largest at the inferior representative level, with a mean value of 31.32 ± 8.36 mm, compared with 23.58 ± 4.57 mm at the mid-level and 22.29 ± 4.35 mm at the superior level. The short axis was 16.71 ± 5.72 mm, 14.85 ± 3.92 mm and 16.83 ± 3.88 mm at the inferior, mid-level and superior levels, respectively.

Area difference was included as a descriptor of deviation between the measured cross-sectional area and the fitted elliptical reference model. Mean area difference was 81.74 ± 104.35 mm^2^ at the inferior level, −19.03 ± 8.08 mm^2^ at the mid-level and −45.59 ± 29.34 mm^2^ at the superior level. These values illustrate that the workflow can quantify not only cross-sectional size, but also the relationship between the observed section outline and its simplified ellipse-based approximation.

Distance-based descriptors further demonstrated the ability of the method to describe local outline geometry. The maximum distance from the ellipse centre was 18.47 ± 5.26 mm at the inferior level, 13.05 ± 2.55 mm at the mid-level and 12.82 ± 2.42 mm at the superior level. The corresponding minimum distances from the ellipse centre were 3.76 ± 2.30 mm, 6.25 ± 1.93 mm and 3.24 ± 3.05 mm, respectively.

Together, these results demonstrate that the semi-automated workflow generated a multi-parameter quantitative description of RVOT geometry. Cross-sectional area was used as the main visual example, whereas ellipse-based and distance-based descriptors showed that the same framework can be extended to shape, outline deviation and local asymmetry analysis.

### 3.5. Methodological ValidationResults

Synthetic testing confirmed the numerical correctness of the implemented geometric calculations under ideal conditions. Cross-sectional area showed a mean absolute percentage error of approximately 0.005%, while ellipse-derived dimensions and major-axis orientation showed negligible numerical error. Robustness analyses indicated that ellipse-derived dimensions were comparatively stable under the tested perturbations, whereas boundary-derived cross-sectional area was more sensitive to coordinate noise, outliers, incomplete contours and within-section geometric variation. Sensitivity analysis of the boundary reconstruction parameter showed that the median absolute change in cross-sectional area relative to the shrink factor of 0.50 was 0.33% for a shrink factor of 0.25 and 0.18% for 0.75. Larger differences occurred only in individual perturbed synthetic contours. The arithmetic-mean centroid showed a median error of 0.025 mm relative to the known centre across all 462 synthetic sections, compared with 0.055 mm for the polygon centroid and 0.069 mm after uniform contour resampling. Larger deviations occurred mainly in deliberately incomplete contours. Uniform arc-length resampling to 120 points had a negligible effect on cross-sectional area, with a median absolute change of 0.052%.

Assessment of the global SVD-based reference axis in 15 RVOT models showed that the first principal component explained a median of 98.52% of centroid positional variance, with a median centroid-to-line RMSE of 1.04 mm, corresponding to 3.76% of the analysed axial RVOT length. For the long dimension, the mean bias was −0.37 mm, with 95% limits of agreement of −4.61 to 3.88 mm and ICC(A,1) of 0.905 (cluster-bootstrap 95% CI: 0.811–0.958). For the short dimension, the mean bias was 0.39 mm, with limits of agreement of −3.09 to 3.87 mm and ICC(A,1) of 0.937 (95% CI: 0.856–0.976). Inter-observer landmark identification showed a mean difference of 0.20 mm and an RMSE of 0.41 mm, with Bland–Altman limits of agreement from −0.52 to 0.92 mm. Detailed results are provided in Supplementary Materials S1.

An additional exploratory inter-observer assessment was conducted on three RVOT models (RVOT-A, RVOT-B, and RVOT-C). Across six processing runs, 68 secondary cross-sections were generated, of which two (2.9%) were excluded due to incomplete wall representation near the superior RVOT boundary. Both observers retained the same number of cross-sections within each model (13, 13, and 7, respectively), yielding 33 paired section-level measurements. Total processing time ranged from 6 min 50 s to 13 min 10 s per model, with longer durations observed during the initial processing runs of each observer. Model-specific section counts and quality-control outcomes are provided in Supplementary Material S1.

The mean of the model-specific RMSE values was 12.97 mm^2^ for cross-sectional area, 0.54 mm for the ellipse major axis, and 0.54 mm for the ellipse minor axis. The corresponding MAE values were 10.63 mm^2^, 0.49 mm, and 0.38 mm, respectively. The mean signed differences (Observer 2 minus Observer 1) were −2.26 mm^2^, +0.49 mm, and −0.11 mm, respectively. The complete descriptive agreement statistics are presented in Table 5.

**Figure 7:**
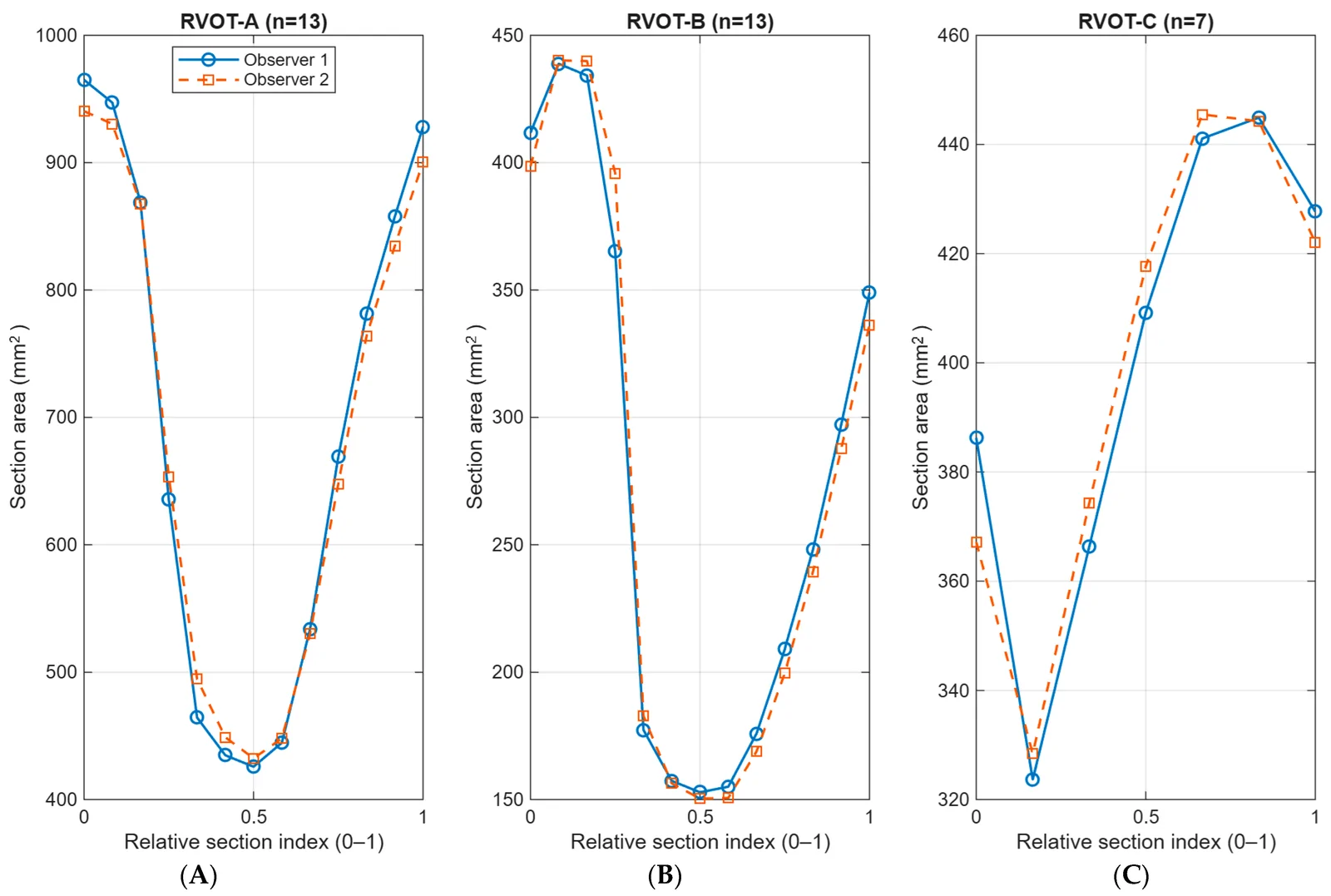
Inter-observer comparison of cross-sectional area profiles in three RVOT models: (**A**) RVOT-A (13 sections), (**B**) RVOT-B (13 sections), and (**C**) RVOT-C (7 sections). Solid blue lines represent Observer 1, and dashed orange lines represent Observer 2. The horizontal axis indicates the normalised sequential position of retained cross-sections (0–1), from the first to the last retained section. The profiles illustrate longitudinal variations in cross-sectional area and local differences between observers.

Comparison of the cross-sectional area profiles (Figure 7) showed broadly similar longitudinal patterns in all three RVOT models, including corresponding regions of local narrowing and widening. Nevertheless, observer-dependent discrepancies were observed at individual section levels, indicating that agreement was not uniform throughout the analysed segments. Additional model-specific agreement statistics and ellipse-based descriptor profiles are presented in Supplementary Materials S1.

## 4. Discussion

### 4.1. Principal Findings and Novelty of the Workflow

The present study addressed a methodological gap between three-dimensional RVOT visualisation and structured quantitative analysis of RVOT geometry. Previous anatomical and reconstruction-based studies have demonstrated the complex spatial morphology and inter-individual variability of the RVOT [5,6,15,16]. Although quantitative analyses of reconstructed RVOT models have also been reported [8], these analyses have primarily focused on selected geometric characteristics, such as the spatial distribution and displacement of cross-sectional centroids. A systematic workflow for representing successive RVOT cross-sections using a multidimensional set of geometric descriptors remains less developed.

The aim of this study was to develop and evaluate a semi-automated workflow for quantitative analysis of RVOT geometry based on 3D point-cloud models. The proposed method combines anatomically guided model preparation, estimation of a model-specific directional line, generation of secondary cross-sections and extraction of geometric descriptors. Its main methodological contribution is that it enables the RVOT to be analysed as a spatially changing structure rather than as a single manually measured dimension.

The workflow was successfully applied to 60 RVOT point-cloud models. After quality control, 661 secondary cross-sections were retained for analysis, with 5–16 sections generated per model. Each retained cross-section was represented by positional, ellipse-based, distance-based and area-based descriptors. These results demonstrate that the workflow can generate a structured, level-based quantitative output from anatomically variable RVOT models.

The present study builds on, but does not reproduce, our previous analysis of the RVOT [8]. Both studies used the same 60 adult human hearts and the same 3D point-cloud models. In [8], the principal objective was to characterise the three-dimensional configuration of the RVOT by analysing the spatial distribution and displacement of cross-sectional centroids, including comparisons between predefined age groups. The computational analysis was therefore centred on centroid coordinates and their relationship to the RVOT axis. In contrast, the present study addresses a different methodological objective: how an existing three-dimensional RVOT point-cloud model can be transformed into a standardised series of cross-sections and subsequently represented by a multidimensional set of geometric descriptors. Here, centroid calculation is used as an intermediate step for estimating the model-specific directional line and standardising the RVOT geometry, rather than as the primary analytical endpoint. The subsequent generation of standardised secondary sections and extraction of ellipse-based, area-based, distance-based and quadrant-based descriptors constitute the principal methodological output of the present work. The age-group comparisons and centroid-displacement findings reported in [8] are therefore not reproduced as the primary results of the present study.

Importantly, this study was methodological in nature. It was not intended to identify clinical subgroups or test associations with clinical variables, but to demonstrate that RVOT point-cloud geometry can be converted into a structured dataset suitable for descriptive geometric assessment. The resulting dataset contains multiple descriptors for each cross-sectional level, allowing different aspects of local RVOT geometry to be examined simultaneously rather than reducing the structure to a single measurement.

Although three-dimensional models provide an anatomically rich representation of complex structures, visualisation alone does not fully exploit the quantitative potential of such data. Recent point-cloud and mesh-based approaches in cardiac and anatomical modelling demonstrate that 3D representations can be used not only for reconstruction and visualisation, but also to model and quantify anatomical shape variation [17,18]. In this context, an additional strength of the proposed workflow is the transformation of complex RVOT point-cloud geometry into a structured quantitative dataset. Such representation facilitates subsequent statistical analysis and may enable integration with computational modelling pipelines and future image-analysis or machine-learning applications [17].

The methodological framework may also provide a basis for future studies comparing RVOT geometry between anatomical or clinical groups, provided that appropriate independent datasets and predefined analytical criteria are used. Because the workflow generates descriptors at multiple standardised relative levels along the RVOT, it may allow future investigations to examine not only overall size but also changes in cross-sectional shape, orientation and local asymmetry along the outflow tract. The present study, however, was intentionally limited to demonstrating the workflow and its quantitative output rather than testing such group differences.

### 4.2. Methodological Value and Interpretation of the Quantitative Output

Quantitative assessment of RVOT geometry is relevant because the outflow tract is anatomically complex, spatially variable and potentially important for anatomical description and imaging interpretation [15,19]. Isolated linear measurements provide information on selected dimensions but do not describe changes in cross-sectional geometry along the entire analysed RVOT segment.

The proposed workflow addresses this issue by generating a sequence of secondary cross-sections along a standardised RVOT segment. The global SVD-based directional line was not intended to represent the anatomical centreline of the RVOT, but to provide a consistent model-specific reference axis for generating mutually parallel cross-sectional planes. This common section orientation was retained deliberately to enable comparison of geometric descriptors, including changes in ellipse orientation, along the analysed segment. Assessment in a subset of 15 RVOT models showed that the first principal component explained a median of 98.52% of centroid positional variance, supporting the use of the global line as a practical reference for the present workflow.

Similar point-cloud modelling strategies have been used for elongated or approximately cylindrical objects, where dense spatial data are reduced to simplified geometric representations such as reference axes, cross-sections or section-based descriptors [11,20]. In the present study, however, the RVOT was not assumed to be cylindrical or geometrically symmetric. The fitted directional line served only as a global reference for model standardisation and generation of secondary cross-sections.

Cross-sectional area was selected as the main representative descriptor for visualisation because it provides an intuitive measure of local RVOT size. The scatter plot with median profile and interquartile range demonstrated that section area can be compared along the relative RVOT level despite differences in the absolute length of the analysed segment between models. However, section area represents only one part of the generated dataset. The same level-based representation can be applied to ellipse-axis measurements, ellipse area, area difference and distance-based descriptors.

The descriptive results for inferior, mid-level and superior representative levels illustrate that the workflow generates descriptors related to different aspects of cross-sectional geometry. Long and short axes describe dominant section dimensions, area difference reflects deviation from the fitted elliptical approximation, and distance-based descriptors provide information about local outline deviations. These parameters should be regarded as quantitative outputs of the workflow rather than validated clinical markers.

The structured descriptor dataset generated by the workflow may also constitute an appropriate input for future machine-learning approaches aimed at automated classification of RVOT morphology, identification of geometric phenotypes and quantitative analysis of inter-individual anatomical variability [13,21]. Such applications, however, require validation in independent datasets.

Rather than representing RVOT geometry using a single measurement at a predefined level, the proposed workflow generates descriptors at multiple relative levels along the analysed segment. This provides a level-based representation of geometric variation along the RVOT. This profile-based representation provides information on geometric variation along the RVOT that is not represented by a single-level measurement and may facilitate identification of local geometric features [15].

### 4.3. Potential Anatomical and Future Procedural Relevance

Although no clinical outcomes or procedural performance were evaluated in the present study, the extracted descriptors were selected to represent geometrically interpretable characteristics of the RVOT. Section area describes local cross-sectional size, ellipse-axis measurements characterise dominant and minor dimensions, area difference reflects deviation from a simplified elliptical representation, and distance-based descriptors describe local outline irregularity. The geometric interpretation of the extracted descriptor groups is summarised in Table 6.

These descriptors should be regarded as methodological outputs rather than validated clinical markers. Their principal value in the present study lies in providing a structured quantitative representation of RVOT geometry at multiple relative levels.

RVOT geometry is clinically relevant in several settings. The RVOT is a common site of origin of idiopathic ventricular arrhythmias, and detailed knowledge of its anatomy is relevant to catheter navigation and electroanatomical mapping [22]. Similarly, assessment of RVOT dimensions and morphology is important in structural interventions involving the outflow tract, including transcatheter pulmonary valve implantation [23]. However, the present study did not evaluate whether the proposed descriptors improve procedural planning, device selection, catheter navigation or clinical outcomes.

Future studies using patient-specific models reconstructed from computed tomography or cardiac magnetic resonance imaging will be required to determine whether the descriptor-based representation developed here provides clinically useful information beyond established imaging measurements. Such studies could also investigate relationships between local RVOT geometry, electroanatomical mapping characteristics and the spatial distribution of arrhythmogenic foci.

The proposed descriptor framework may additionally facilitate future comparisons between post-mortem anatomical specimens and imaging-derived three-dimensional models by expressing RVOT geometry using a common set of quantitative parameters. Whether such an approach improves comparability across imaging modalities, reconstruction methods or research centres remains to be established.

### 4.4. Limitations and Future Directions

This study has several limitations. The analysis was based on post-mortem anatomical material and silicone casts; therefore, the models represented static RVOT geometry and did not capture dynamic in vivo changes during the cardiac cycle. Point-cloud quality also depended on casting, photogrammetric reconstruction and model-cleaning steps. Local artefacts or incomplete surface representation could affect generated cross-sections, which is why visual quality control was required, particularly for terminal sections.

The anatomical sample was also sex-imbalanced, comprising 45 male and 15 female hearts. Because the present study was designed as a methodological proof of concept rather than a sex-stratified morphometric analysis, sex-specific differences in RVOT geometry were not evaluated. Consequently, the descriptive geometric distributions reported in this study should not be interpreted as representative of sex-specific or population-level RVOT morphology. Future studies using larger and more balanced datasets are required to determine whether the extracted geometric descriptors differ systematically between male and female hearts.

Although the silicone casting procedure was designed to minimise mechanical deformation of the cardiac structures, the potential effects of fixation, silicone polymerisation, and cast extraction on the resulting geometry were not quantitatively assessed. Furthermore, while photogrammetric scale calibration was performed using physical reference measurements, the residual geometric reconstruction error was not independently quantified.

The workflow was semi-automated rather than fully automated. Manual input was required for RVOT point-cloud preparation, anatomical landmark identification, and quality control of the generated sections. Inter-observer variability in anatomical landmark identification and agreement between ellipse-derived dimensions and independent manual measurements were assessed. An additional exploratory inter-observer assessment of geometric descriptors was conducted using three RVOT models. The results demonstrated broadly similar longitudinal geometric profiles, although local discrepancies between observers were observed. The limited number of independent anatomical specimens (*n* = 3) restricts the generalisability of these findings. Although section-exclusion decisions and total processing times were documented, exact spatial correspondence between paired sections was not independently verified. Manual and computational times were not recorded separately, and the number of manual interventions was not systematically quantified. Intra-observer repeatability was not assessed, and the manual measurements represent an independent reference rather than an anatomical gold standard. Consequently, the present findings should not be interpreted as comprehensive validation of whole-workflow reproducibility.

The present workflow provides longitudinal but not rotational anatomical standardisation of the RVOT models. Alignment of the model-specific directional axis establishes a common longitudinal reference but does not define rotation around this axis. Consequently, ellipse orientation and quadrant labels are defined within the local geometric coordinate system of each cross-section and should not be interpreted as corresponding to fixed anatomical directions across different models. In addition, the major axis of the fitted ellipse is intrinsically undirected and therefore has a 180° orientation ambiguity. The quadrant-based descriptors should thus be interpreted as local geometric measures rather than anatomically registered regional measurements. Future development of the workflow should incorporate a second reproducible anatomical landmark or another rotational registration procedure to establish a common anatomical coordinate system across RVOT models.

No hypothesis testing or clinical subgroup comparison was performed, as the study was methodological in nature. Future investigations should include a larger inter- and intra-observer repeatability study, with explicit evaluation of anatomical boundary placement, section-exclusion decisions, processing time, and the number of manual interventions. Further validation against independent reference standards and imaging-derived RVOT models is also warranted.

In summary, the proposed semi-automated workflow addresses the methodological gap between 3D RVOT visualisation and structured quantitative geometric analysis.

It provides a semi-automated method for converting complex RVOT point-cloud geometry into a level-based set of descriptors describing local size, shape, outline deviation and asymmetry. By extending previous anatomical and clinical observations on the importance of RVOT morphology, this workflow creates a quantitative basis for future studies linking RVOT geometry with imaging interpretation, anatomical variability and procedure-related planning. Further work should therefore focus on repeatability assessment, validation in imaging-derived models and translation of the descriptor-based approach into clinically oriented RVOT analyses.

## 5. Conclusions

The present study introduces a semi-automated workflow for transforming 3D RVOT point-cloud models into a structured, level-based dataset of geometric descriptors. The workflow was successfully applied to 60 anatomical RVOT models and generated 687 secondary cross-sections, of which 661 were retained after quality control for quantitative description of local cross-sectional size, shape and outline variation.

Complementary validation supported the numerical correctness of the implemented geometric calculations, the use of the global SVD-based line as a model-specific longitudinal reference, agreement between ellipse-derived dimensions and independent manual measurements, and low inter-observer variability in anatomical landmark identification. The validation also demonstrated that boundary-derived cross-sectional area is more sensitive than ellipse-based descriptors to noise, outliers, incomplete contours and within-section geometric variation.

The proposed workflow should therefore be regarded as a methodological proof of concept for standardised quantitative description of RVOT geometry. The global SVD-based line represents a geometric reference rather than a true anatomical centreline, and the present validation does not constitute clinical or anatomical gold-standard validation of the complete workflow. Further studies should address intra-observer repeatability and validation in independent and imaging-derived RVOT datasets before clinical applicability can be established. An exploratory inter-observer comparison involving three RVOT models showed generally comparable longitudinal profiles of cross-sectional geometry, with measurable local differences in the extracted descriptors. These preliminary findings provide an initial assessment of inter-observer agreement but do not establish comprehensive repeatability of the entire workflow.

## Figures and Tables

**Table 1:** Comparison of the previously published analysis [8] and the present methodological study.

Element	Previous Study [8]	Present Study
Anatomical material	60 adult human hearts	The same 60 adult human hearts
3D data	RVOT 3D point-cloud models derived from silicone casts and photogrammetric reconstruction	The same existing RVOT 3D point-cloud models
General objective	Anatomical analysis of the three-dimensional configuration of the RVOT	Development and methodological evaluation of a semi-automated workflow for level-based quantitative analysis of RVOT geometry
Cross-section selection	Cross-sections were manually selected for measurement and anatomical analysis	Primary cross-sections are prepared for directional-reference estimation, followed by systematic generation of consecutive secondary analytical cross-sections
Automated sequential section generation	Not performed	Introduced in the present workflow; consecutive 3-mm secondary cross-sections are generated computationally along the model-specific directional reference
Primary-section centroids	Centroid position and displacement were principal variables used to characterise the spatial configuration of the RVOT	Centroids of the primary cross-sections are used as intermediate geometric information to estimate the model-specific global directional reference using SVD
Secondary-section centroids	No automated sequential secondary-section analysis was performed	The centroid of each retained secondary cross-section is calculated as one of several local geometric descriptors and is also used as a reference for distance-based measurements
Geometric standardisation	Not the principal methodological objective of the study	A model-specific global directional reference and mathematically defined inferior boundary are used to standardise section generation along the analysed RVOT segment
Measurements/descriptors	Manually selected cross-sections and centroid-based morphometric analysis	Computational extraction of positional, ellipse-based, area-based, distance-based and local geometric descriptors from each retained secondary cross-section
Longitudinal representation of the RVOT	Analysis based on manually selected cross-sectional locations	Consecutive analytical sections are represented according to their normalised position along the analysed RVOT segment
Group analysis	Included assessment of centroid displacement and comparisons between predefined age groups	No age-group or other clinical subgroup comparisons; the analysis focuses on methodological performance and geometric descriptor generation
Methodological validation	Not the primary objective of the previous study	Synthetic robustness testing, assessment of the global SVD-based reference, comparison with independent manual measurements and observer-related validation
Principal output	Anatomical findings describing RVOT configuration and centroid displacement	A semi-automated processing workflow and structured, level-based multi-parameter dataset describing RVOT geometry
Previously published results reused as study outcomes	—	Previously reported centroid-displacement and age-group findings are not reused as outcomes of the present study

**Table 2:** Geometric descriptors extracted from each secondary RVOT cross-section.

Descriptor Group	Descriptor	Symbol	Definition/Calculation	Unit
Section identifier	Section number	*k*	Consecutive number of the secondary cross-section from the inferior to the superior RVOT boundary	–
Position	Section centroid	c*_S,k_*	Arithmetic mean of the projected points belonging to the (*k*)-th secondary cross-section	mm
Ellipse-based	Ellipse centre	c*_E,k_*	Centre of the ellipse fitted to the section points	mm
Ellipse-based	Ellipse semi-axis	*a_k_*	Ellipse semi-axis of the fitted ellipse	mm
Ellipse-based	Ellipse semi-axis	*b_k_*	Ellipse semi-axis of the fitted ellipse	mm
Ellipse-based	Long axis	*2max*(*a_k,_ b_k_*)	Length of the major axis of the fitted ellipse	mm
Ellipse-based	Short axis	*2min*(*a_k,_ b_k_*)	Length of the minor axis of the fitted ellipse	mm
Ellipse-based	Ellipse orientation	*θ_k_*	Orientation angle of the fitted ellipse	radians
Distance-based	Maximum distance from section centroid	$d_{max,k}^{S}$	Maximum distance between the section centroid and points of the section	mm
Distance-based	Minimum distance from section centroid	$d_{min,k}^{S}$	Minimum distance between the section centroid and points of the section	mm
Distance-based	Maximum distance from ellipse centre	$d_{max,k}^{E}$	Maximum distance between the ellipse centre and points of the section	mm
Distance-based	Minimum distance from ellipse centre	$d_{min,k}^{E}$	Minimum distance between the ellipse centre and points of the section	mm
Area-based	Section area	*A_S,k_*	Area enclosed by the reconstructed boundary of the secondary cross-section	mm^2^
Area-based	Ellipse area	*A_E,k_*	Area of the fitted ellipse, *A_E,k_* = *π a_k_ b_k_*	mm^2^
Area-based	Area difference	Δ *A_k_*	Difference between ellipse area and section area, Δ *A_k_* = *A_E,k_* − *A_S,k_*	mm^2^
Local asymmetry	Quadrant I area	*A_Q1,k_*	Area of the first quadrant defined by the fitted ellipse axes	mm^2^
Local asymmetry	Quadrant II area	*A_Q2,k_*	Area of the second quadrant defined by the fitted ellipse axes	mm^2^
Local asymmetry	Quadrant III area	*A_Q3,k_*	Area of the third quadrant defined by the fitted ellipse axes	mm^2^
Local asymmetry	Quadrant IV area	*A_Q4,k_*	Area of the fourth quadrant defined by the fitted ellipse axes	mm^2^

**Table 3:** Quantitative output of the semi-automated RVOT workflow.

Parameter	Value
Analysed RVOT models	60
Secondary cross-sections generated	687
Secondary cross-sections excluded during quality control	26 (3.8%)
Secondary cross-sections retained for quantitative analysis	661
Retained secondary cross-sections per model	5–16
Mean ± SD retained secondary cross-sections per model	11.02 ± 2.47
Median secondary cross-sections per model	11

**Table 4:** Descriptive statistics for selected representative RVOT descriptors at inferior, middle and superior levels.

Descriptor	Inferior Level, Mean ± SD	Mid-Level, Mean ± SD	Superior Level, Mean ± SD
Section area [mm^2^]	356.55 ± 190.40	302.72 ± 121.71	350.50 ± 138.84
Ellipse area [mm^2^]	438.29 ± 247.37	283.69 ± 118.12	304.91 ± 125.61
Area difference [mm^2^]	81.74 ± 104.35	−19.03 ± 8.08	−45.59 ± 29.34
Long axis [mm]	31.32 ± 8.36	23.58 ± 4.57	22.29 ± 4.35
Short axis [mm]	16.71 ± 5.72	14.85 ± 3.92	16.83 ± 3.88
Maximum distance from ellipse centre [mm]	18.47 ± 5.26	13.05 ± 2.55	12.82 ± 2.42
Minimum distance from ellipse centre [mm]	3.76 ± 2.30	6.25 ± 1.93	3.24 ± 3.05

**Table 5:** Exploratory inter-observer agreement in geometric descriptors obtained from three RVOT models.

Geometric Descriptor	Mean Bias	MAE	RMSE
Cross-sectional area (mm^2^)	−2.26	10.63	12.97
Ellipse major axis (mm)	+0.49	0.49	0.54
Ellipse minor axis (mm)	−0.11	0.38	0.54
Ellipse area (mm^2^)	−0.60	12.38	16.94
Area difference (mm^2^)	+1.66	5.07	8.10

Note: Bias represents the signed difference between Observer 2 and Observer 1. MAE denotes mean absolute error, and RMSE denotes root mean square error. Values are arithmetic means of model-specific estimates, with equal weight assigned to each of the three RVOT models (*n* = 3). A total of 33 paired cross-sections were analysed.

**Table 6:** Geometric interpretation of the RVOT descriptors generated by the proposed workflow.

Methodological Element/Descriptor Group	Geometric Interpretation
Mathematically defined inferior RVOT boundary	Defines the inferior limit of the analysed RVOT segment and provides a common starting reference for section generation.
Section number and relative position	Defines the location of each cross-section along the standardised RVOT segment.
Section centroid and ellipse centre	Describe the geometric centres derived from the section point distribution and fitted ellipse, respectively.
Long and short axes	Describe the dominant and minor dimensions of the fitted elliptical approximation of the cross-section.
Ellipse orientation	Describes the orientation of the major axis within the local coordinate system; it does not represent a fixed anatomical direction across models.
Section area	Quantifies the area enclosed by the reconstructed cross-sectional boundary.
Ellipse area and area difference	Describe the area of the fitted elliptical approximation and its difference from the reconstructed section area.
Maximum and minimum distances from the centroid or ellipse centre	Quantify radial extremes of the section relative to the selected geometric centre.
Quadrant areas	Describe the distribution of cross-sectional area within the local ellipse-based coordinate system; individual quadrants do not represent corresponding anatomical regions across models.
Complete descriptor profile	Provides a structured, level-based numerical representation of cross-sectional RVOT geometry along the analysed segment.

## Data Availability

The anatomical material comprising 60 hearts and the source 3D RVOT point-cloud models were previously described in reference [8]. The present study introduces a new semi-automated analytical workflow and generates a distinct dataset of secondary cross-sections and geometric descriptors, together with methodological validation specific to this work.

## Supplementary Materials

The following supporting information can be downloaded at: https://www.mdpi.com/article/10.3390/s26217037/s1.
