# Supplementary material for: A Semi-Automated Workflow for Quantitative Analysis of Right Ventricular Outflow Tract Geometry Based on 3D Point-Cloud Models
Source: Sensors (Basel). 2026 Nov 6;26(21):7037. doi: 10.3390/s26217037 (PMC13766451; doi:10.3390/s26217037)
Supplement: Supplementary file 1 [file sensors-26-07037-s001.zip › Supplementary Material S1_v2.pdf]

sensors

MDPI

Supplementary Material S1 – Validation of the RVOT analysis workflow

S1.1. Synthetic geometric validation and robustness assessment

Synthetic cross-sections with predefined geometries were used to verify the numerical correctness and robustness of the computational procedure independently of anatomical variability. The reference dataset comprised a circle and ellipses with known centre coordinates, semi-axis dimensions and orientations. Under ideal conditions, the workflow reproduced the ellipse dimensions, centre position and major-axis orientation with negligible numerical error. The mean absolute percentage error for cross-sectional area was approximately 0.005%, attributable to polygonal discretisation of the synthetic contour.

Robustness was subsequently evaluated using 462 synthetic cross-sections derived from two representative elliptical geometries. For each geometry, 20 realisations were generated at each level of Gaussian noise (SD = 0.05, 0.10, 0.25 and 0.50 mm) and outlier contamination (1%, 3% and 5%). Ten realisations per geometry were generated at each level of missing contour (5%, 10%, 20% and 30%) and point density (30, 60, 120, 240 and 480 contour points). Two unperturbed control sections were also included. The controlled perturbations therefore comprised coordinate noise, randomly distributed outliers, incomplete contours and variation in point density. Coordinate noise progressively affected boundary-derived area, with the median absolute relative area error increasing from approximately 1.1% at a noise standard deviation of 0.05 mm to 16.2% at 0.50 mm. In contrast, the ellipse-derived long and short axes and major-axis orientation remained comparatively stable. Outliers produced a similar pattern: at 5% contamination, the median absolute relative area error reached approximately 35%, whereas errors in the fitted axes remained below 2%. Incomplete contours had a strongly location-dependent effect on area estimation; with 30% of the contour missing, the median absolute relative area error was approximately 22%, and the largest errors exceeded 50%. Reducing point density had little effect at moderate and high densities, whereas the sparsest synthetic sections showed increased area error.

A separate deterministic sensitivity test examined geometric variation within the 3-mm section thickness at four levels of lateral displacement (0.5, 1, 2 and 3 mm) and three levels of dimensional change (2%, 5% and 10%). Increasing lateral displacement or dimensional change through the section primarily affected boundary-derived area, whereas ellipse-based dimensions and orientation were substantially less sensitive.

An additional sensitivity analysis evaluated the influence of the boundary reconstruction parameter using the same 462 synthetic cross-sections. For each section, the boundary-derived area was recalculated using MATLAB boundary shrink factors of 0.25, 0.50 and 0.75, while the underlying point set and all other processing steps remained unchanged. A shrink factor of 0.50 corresponded to the value used in the principal workflow. Across all synthetic sections, the median absolute change in cross-sectional area relative to the 0.50 setting was 0.33% for a shrink factor of 0.25 and 0.18% for 0.75. The corresponding median absolute area errors relative to the known ground-truth geometry were 5.47%, 6.00% and 5.47% for shrink factors of 0.25, 0.50 and 0.75, respectively. Occasional larger differences between settings occurred in perturbed contours, indicating increased parameter sensitivity when boundary quality was degraded.

Sensitivity to centroid definition and contour resampling was assessed using the same synthetic dataset. The arithmetic-mean centroid used in the principal workflow was compared with the area centroid of the reconstructed boundary polygon and with the centroid obtained after uniform arc-length resampling to 120 contour points. The median error relative to the known centre was 0.025 mm for the arithmetic-mean centroid, 0.055 mm for the polygon centroid and 0.069 mm after uniform resampling. Larger deviations occurred mainly in deliberately incomplete contours. Uniform resampling had a negligible effect on boundary-derived area, with a median absolute change of 0.052%.

These experiments confirmed the numerical correctness of the implementation under ideal geometric conditions and demonstrated that the derived descriptors have different robustness characteristics. Ellipse-based measurements were comparatively stable under the tested perturbations, whereas boundary-derived cross-sectional area was more sensitive to noise, outliers, incomplete contours and through-thickness geometric variation. The validation design and principal robustness results are summarised in Table S1.

Table S1. Synthetic geometric validation and robustness assessment of the RVOT cross-sectional analysis workflow

Validation component	Test conditions / perturbation levels	n	Cross-sectional area	Ellipse-derived dimensions	Major-axis orientation	Main observation
Ideal geometry	Circle and four predefined ellipses	5	MAPE ≈ 0.005%	Negligible numerical error	Negligible numerical error	Numerical correctness confirmed
Gaussian coordinate noise	SD = 0.05 / 0.10 / 0.25 / 0.50 mm	160	Median error: 1.14 / 2.48 / 6.78 / 16.15%	Remained low; at 0.50 mm: long ≈ 0.28%, short ≈ 1.39%	≈ 0.12° at 0.50 mm	Area progressively sensitive to noise
Random outliers	1 / 3 / 5%	120	Median error: 10.84 / 26.27 / 34.97%	At 5%: long ≈ 0.69%, short ≈ 1.73%	≈ 0.12° at 5%	Area highly sensitive; ellipse descriptors comparatively stable
Incomplete contour	Missing arc = 5 / 10 / 20 / 30%	80	Median error: 0.14 / 0.83 / 14.85 / 22.05%	Minimal under ideal remaining-contour conditions	Minimal under ideal remaining-contour conditions	Area strongly dependent on extent and location of missing contour
Point density	30 / 60 / 120 / 240 / 480 points	100	Error increased mainly at the lowest densities; ≤0.02% median at ≥120 points*	Stable at moderate/high density	Stable	Sparse contours mainly affected boundary-derived area
Boundary shrink factor	0.25 / 0.50 / 0.75	462	Median absolute change vs 0.50: 0.33% / 0.18%	Not affected	Not affected	Limited influence under typical contour conditions

Centroid definition / contour resampling	Arithmetic mean / polygon centroid / 120-point uniform resampling	462	Median absolute area change after resampling: 0.052%	-	-	Median centre error: 0.025 / 0.055 / 0.069 mm
Through-thickness lateral displacement	0.5 / 1 / 2 / 3 mm within a 3-mm section	separate deterministic test	Error increased to ≈ 12.8% at 3 mm displacement	At 3 mm: long ≈ 1.53%, short ≈ 0.19%	≈ 0.30°	Boundary area more sensitive than ellipse descriptors
Through-thickness dimensional change	2 / 5 / 10%	separate deterministic test	Error ≈ 2.0 / 5.1 / 10.2%	comparatively stable	comparatively stable	Area reflected geometric variation within section thickness

The five ideal geometries were evaluated in a separate numerical-correctness test, whereas the 462-section Monte Carlo dataset included two additional unperturbed control sections.

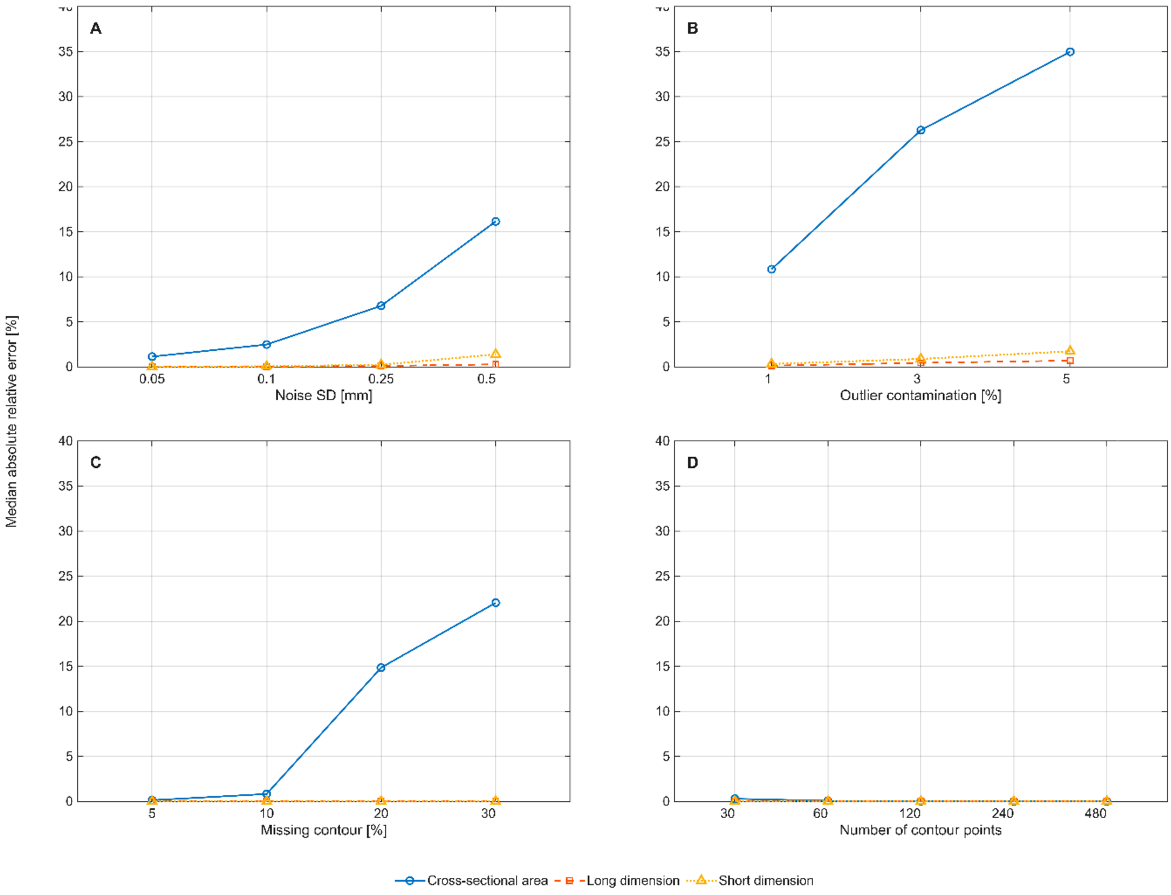

Figure S1. Robustness of the derived cross-sectional descriptors under controlled synthetic perturbations. Median absolute relative errors are shown for boundary-derived cross-sectional area and ellipse-derived long and short dimensions under increasing coordinate noise (A), outlier contamination (B), incomplete contour (C), and variation in point density (D).

S1.2. Assessment of the global SVD-based reference axis

The suitability of the global SVD-based directional line was assessed in a subset of 15 RVOT models, comprising 224 primary-section centroids (10–19 primary sections per model). The models were selected without applying model-specific selection criteria or inspecting model geometry or validation results. The line was not intended to represent an anatomical centreline but to provide a model-specific reference axis for generating mutually parallel secondary cross-sections. Primary-section centroids were spatially ordered along the SVD direction, and their orthogonal distances from the fitted line were calculated. Model-level assessments included centroid-to-line RMSE, RMSE normalised by axial RVOT length, variance explained by the first principal component, centroid-path tortuosity and the angular deviation between local centroid directions and the global SVD axis.

Across the 15 models, the first principal component explained a median of 98.52% of the variance in centroid position (IQR 96.79–98.90%). The median centroid-to-line RMSE was 1.04 mm (IQR 0.82–1.64 mm), corresponding to 3.76% (IQR 3.25–5.60%) of the analysed axial RVOT length. Larger deviations occurred in more curved RVOT geometries, confirming that the global line represents a deliberate geometric simplification rather than a local anatomical trajectory. Nevertheless, the assessment supported its use as a common directional reference for standardised cross-section generation and comparison of geometric descriptors along the RVOT. Summary results for the assessment of the global SVD-based reference axis are provided in Table S2.

Table S2. Summary assessment of the global SVD-based reference axis

Metric	Summary value
Number of RVOT models	15
Number of primary-section centroids	224
PCA1 explained variance [%]	98.52 [96.79–98.90]
Centroid-to-line RMSE [mm]	1.04 [0.82–1.64]
Normalised RMSE [% of axial length]	3.76 [3.25–5.60]
Tortuosity	1.053 [1.037–1.123]
Median local-to-global angle [°]	11.30 [9.50–13.76]
Maximum local-to-global angle [°]	19.62 [15.89–26.90]

Values are presented as median [IQR], except for the number of models and centroids.

S1.3. Agreement between manual and ellipse-derived dimensions

Agreement between the automated geometric descriptors and independent manual measurements was evaluated using 45 secondary cross-sections obtained from the same 15 RVOT models, with three sections analysed per model. For each RVOT model, one cross-section was selected from each of the inferior, middle and superior portions of the analysed segment without prior inspection of section geometry or automated measurement results. This yielded 45 paired comparisons for the long dimension and 45 for the short dimension. Manual measurements were performed without access to the corresponding automated results. For each section, the operator identified the largest visually apparent cross-sectional dimension and then measured the perpendicular short dimension through its midpoint. These measurements were compared with the long and short axes derived from the fitted ellipse. CC confidence intervals were estimated using 10,000 heart-clustered bootstrap resamples, with all three sections from each resampled heart retained together.

For the long dimension, the mean bias (ellipse-derived minus manual) was −0.37 mm, with an MAE of 1.49 mm and an RMSE of 2.17 mm. The absolute-agreement ICC(A,1) was 0.905, and the Bland–Altman limits of agreement were −4.61 to 3.88 mm. For the short dimension, the mean bias was 0.39 mm, with an MAE of 1.13 mm and an RMSE of 1.80 mm. The corresponding ICC(A,1) was 0.937, with limits of agreement of −3.09 to 3.87 mm.

Larger discrepancies were observed primarily in highly irregular cross-sections, in which a manually selected local diameter and the axis of a globally fitted ellipse represent different geometric characteristics. Accordingly, ellipse fitting was treated as a standardised descriptor of cross-sectional geometry rather than as an assumption that the anatomical contour was intrinsically elliptical. Summary agreement statistics are provided in Table S3, and the corresponding scatter and Bland–Altman plots are shown in Figure S2.

Table S3. Agreement between manual and ellipse-derived RVOT cross-sectional dimensions

Dimension	n	Mean bias [mm]	MAE [mm]	RMSE [mm]	ICC(A,1)	95% limits of agreement [mm]	95% CI for ICC(A,1)
Long dimension	45	−0.37	1.49	2.17	0.905	−4.61 to 3.88	0.811–0.958
Short dimension	45	0.39	1.13	1.80	0.937	−3.09 to 3.87	0.856–0.976

Manual measurements represent independent reference measurements of visually identified local dimensions, whereas automated values correspond to the axes of the globally fitted ellipse.

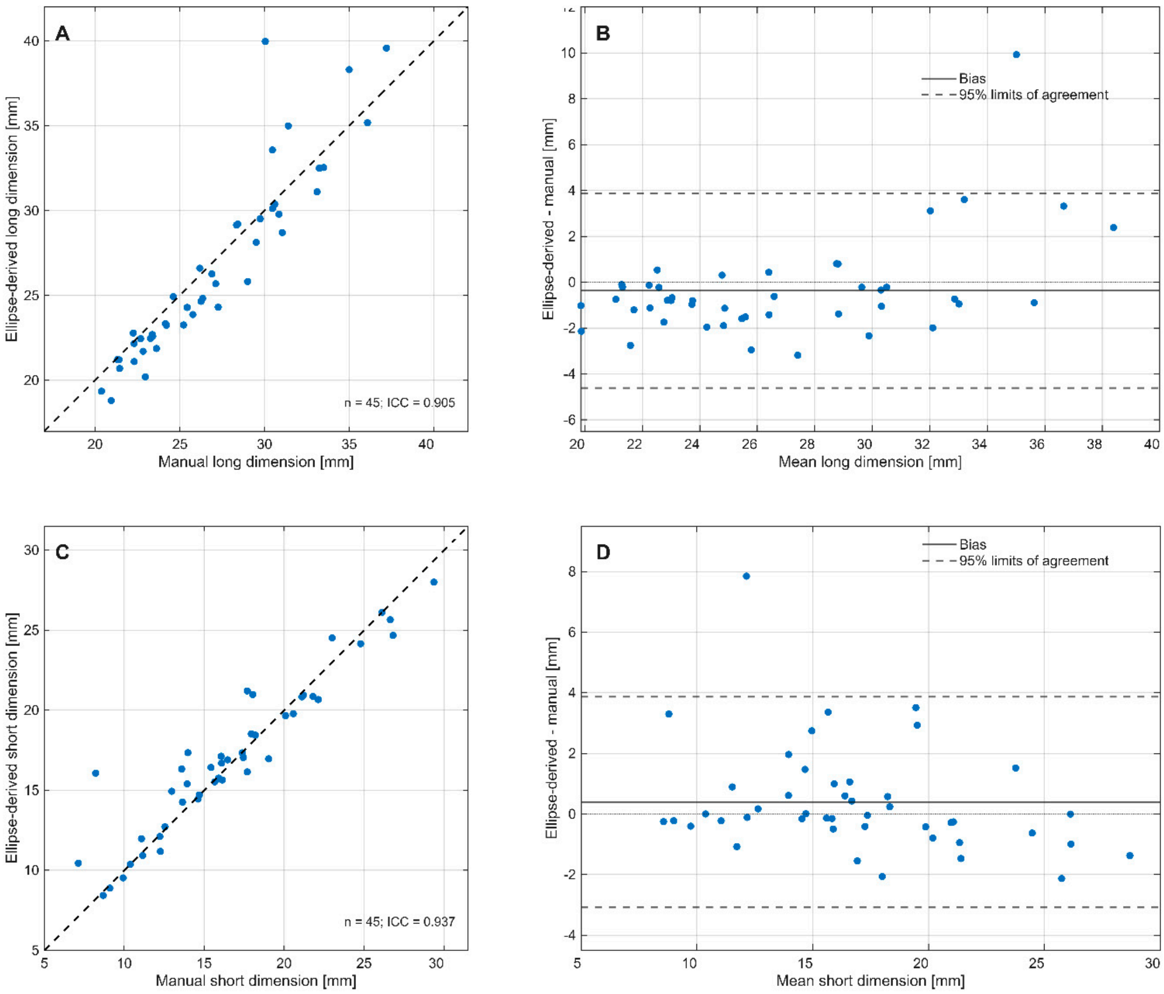

Figure S2. Agreement between manual reference measurements and ellipse-derived cross-sectional dimensions. Scatter plots with the identity line are shown for the long (A) and short (C) dimensions, with corresponding Bland–Altman plots for the long (B) and short (D) dimensions.

S1.4. Inter-observer agreement in anatomical landmark identification

Inter-observer agreement was assessed in the same 15 RVOT models by comparing independent landmark selections made by two operators. Each operator identified the anatomical landmark once in each model, yielding 30 landmark selections and 15 paired inter-observer comparisons. Because the transformed coordinate system defines a fixed zero plane and the selected landmark determines the height of the inferior RVOT boundary plane, agreement was evaluated using the vertical coordinate Z.

The mean inter-observer difference in landmark height (Operator 2 − Operator 1) was 0.20 mm (SD 0.37 mm), with an MAE of 0.30 mm and an RMSE of 0.41 mm. The median absolute difference was 0.26 mm (IQR 0.12–0.35 mm), whereas the 95th percentile and maximum absolute differences were 0.81 and 1.11 mm, respectively. The Bland–Altman limits of agreement were −0.52 to 0.92 mm. The absolute-agreement ICC(A,1) was 1.000 (bootstrap 95% CI 0.999–1.000). Because absolute Z coordinates are model-specific, difference-based metrics were treated as the primary assessment of inter-observer agreement, whereas ICC(A,1) was reported as a secondary measure.

These results indicate that operator-dependent variability in defining the height of the inferior boundary plane was small relative to the analysed RVOT geometry. Summary statistics for inter-observer agreement are provided in Table S4, and the corresponding difference plot is shown in Figure S3.

Table S4. Inter-observer agreement in anatomical landmark height

Metric	Value
Number of RVOT models	15
Mean bias, ΔZ [mm]	0.20
SD of differences [mm]	0.37
MAE [mm]	0.30
RMSE [mm]	0.41
Median absolute difference [mm]	0.26
IQR of absolute difference [mm]	0.12–0.35
95th percentile of absolute difference [mm]	0.81
Maximum absolute difference [mm]	1.11
Bland–Altman 95% limits of agreement [mm]	−0.52 to 0.92
ICC(A,1)	1.000
Bootstrap 95% CI for ICC	0.999–1.000

Agreement was assessed using the Z coordinate because the selected landmark defines the height of the inferior RVOT boundary plane relative to the model-specific fixed zero plane.

The same subset of 15 RVOT models was used for the real-data validation procedures described in Sections S1.2–S1.4. These 15 models were selected from the full dataset of 60 RVOT models without applying model-specific selection criteria or inspecting model geometry or validation results. No formal random-number generator was used.

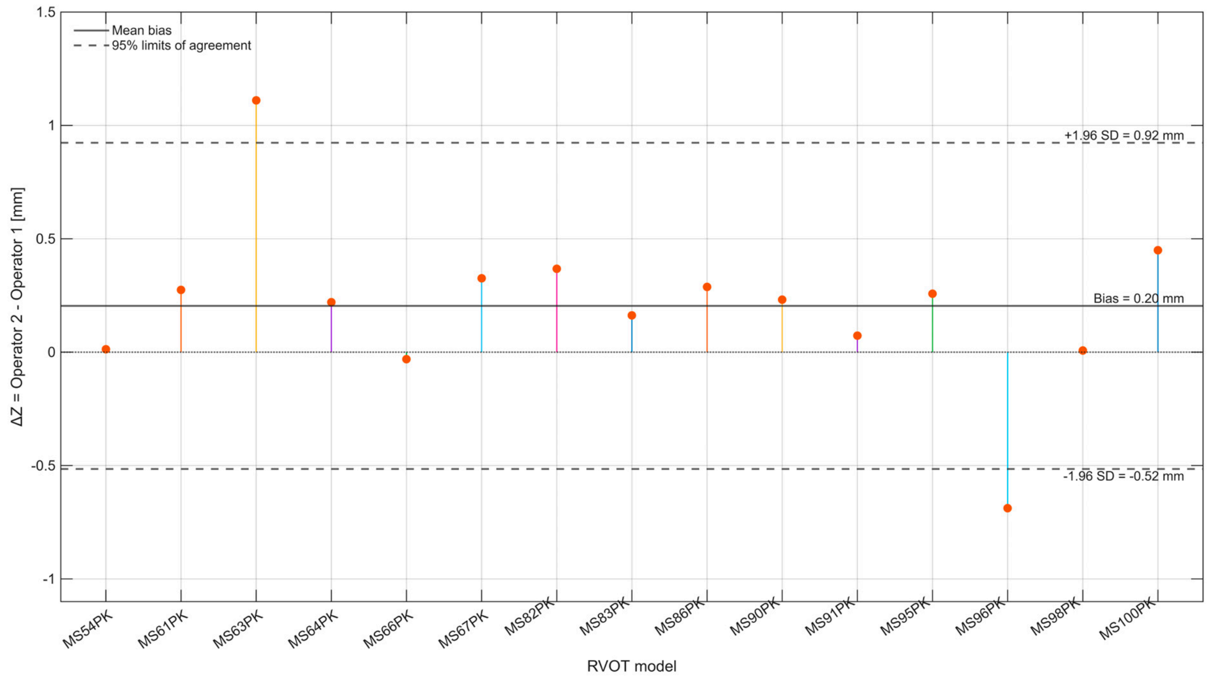

Figure S3. Inter-observer differences in anatomical landmark height across 15 RVOT models. The solid line indicates the mean inter-observer difference, the dashed lines indicate the 95% limits of agreement, and the dotted line represents zero difference.

S1.5. Exploratory inter-observer agreement in geometric descriptors

An exploratory inter-observer assessment was performed using three RVOT models (RVOT-A, RVOT-B, and RVOT-C), independently processed by two observers. The analysis included 33 paired cross-sections (13, 13, and 7 per model, respectively). Agreement was evaluated for cross-sectional area, ellipse major and minor axes, ellipse area, and the difference between cross-sectional and ellipse areas.

Across the six processing runs, 68 secondary cross-sections were generated, of which two (2.9%) were excluded during quality control. One section was excluded by Observer 2 in RVOT-A and one by Observer 1 in RVOT-B. Both exclusions involved incomplete wall representation near the superior RVOT boundary, associated with the inclination of the local RVOT geometry relative to the sectioning planes. No sections were excluded in RVOT-C. Total processing time ranged from 6 min 50 s to 13 min 10 s per model, with the longest durations recorded during the initial processing runs of each observer. Detailed section counts are presented in Table S5.

Table S5. Section generation and quality-control outcomes in the exploratory inter-observer assessment.

RVOT model	Observer	Generated	Excluded	Retained
RVOT-A	1	13	0	13
RVOT-A	2	14	1	13
RVOT-B	1	14	1	13
RVOT-B	2	13	0	13
RVOT-C	1	7	0	7
RVOT-C	2	7	0	7
Total		68	2	66

Note: Excluded sections contained incomplete representations of the RVOT wall near the superior boundary. Processing times ranged from 6 min 50 s to 13 min 10 s per model.

Corresponding sections were paired by their sequential indices, normalised to the interval [0, 1]. Inter-observer differences were quantified using bias (Observer 2 minus Observer 1), mean absolute error (MAE), and root mean square error (RMSE). Statistics were calculated separately for each model to account descriptively for the nested data structure.

Model-specific results are presented in Table S6. Cross-sectional area RMSE ranged from 9.05 to 18.53 mm^2, whereas RMSE for the ellipse major and minor axes ranged from 0.17 to 0.89 mm and from 0.40 to 0.69 mm, respectively. The longitudinal profiles of ellipse dimensions showed broadly similar patterns between observers, although local differences were observed (Figure S4).

Table S6. Model-specific exploratory inter-observer agreement in RVOT geometric descriptors.

RVOT model	Geometric descriptor	n	Bias	MAE	RMSE
RVOT-A	Cross-sectional area	13	−4.85	16.01	18.53
RVOT-A	Ellipse major axis	13	+0.45	0.46	0.58
RVOT-A	Ellipse minor axis	13	−0.14	0.41	0.51
RVOT-A	Ellipse area	13	+1.31	16.59	22.44
RVOT-A	Area difference	13	+6.17	7.75	10.74
RVOT-B	Cross-sectional area	13	−1.95	8.58	11.33
RVOT-B	Ellipse major axis	13	+0.86	0.86	0.89
RVOT-B	Ellipse minor axis	13	+0.07	0.29	0.40
RVOT-B	Ellipse area	13	+0.05	9.42	13.68

RVOT-B	Area difference	13	+2.00	2.98	3.89
RVOT-C	Cross-sectional area	7	+0.03	7.29	9.05
RVOT-C	Ellipse major axis	7	+0.15	0.15	0.17
RVOT-C	Ellipse minor axis	7	−0.25	0.45	0.69
RVOT-C	Ellipse area	7	−3.16	11.11	14.68
RVOT-C	Area difference	7	−3.19	4.48	9.68

Note: Bias was calculated as Observer 2 minus Observer 1. MAE, mean absolute error; RMSE, root mean square error. Area-based descriptors are expressed in mm² and ellipse-axis dimensions in mm. The analysis included three independent RVOT models and 33 paired cross-sections. Each model was analysed separately to account descriptively for the nested data structure.

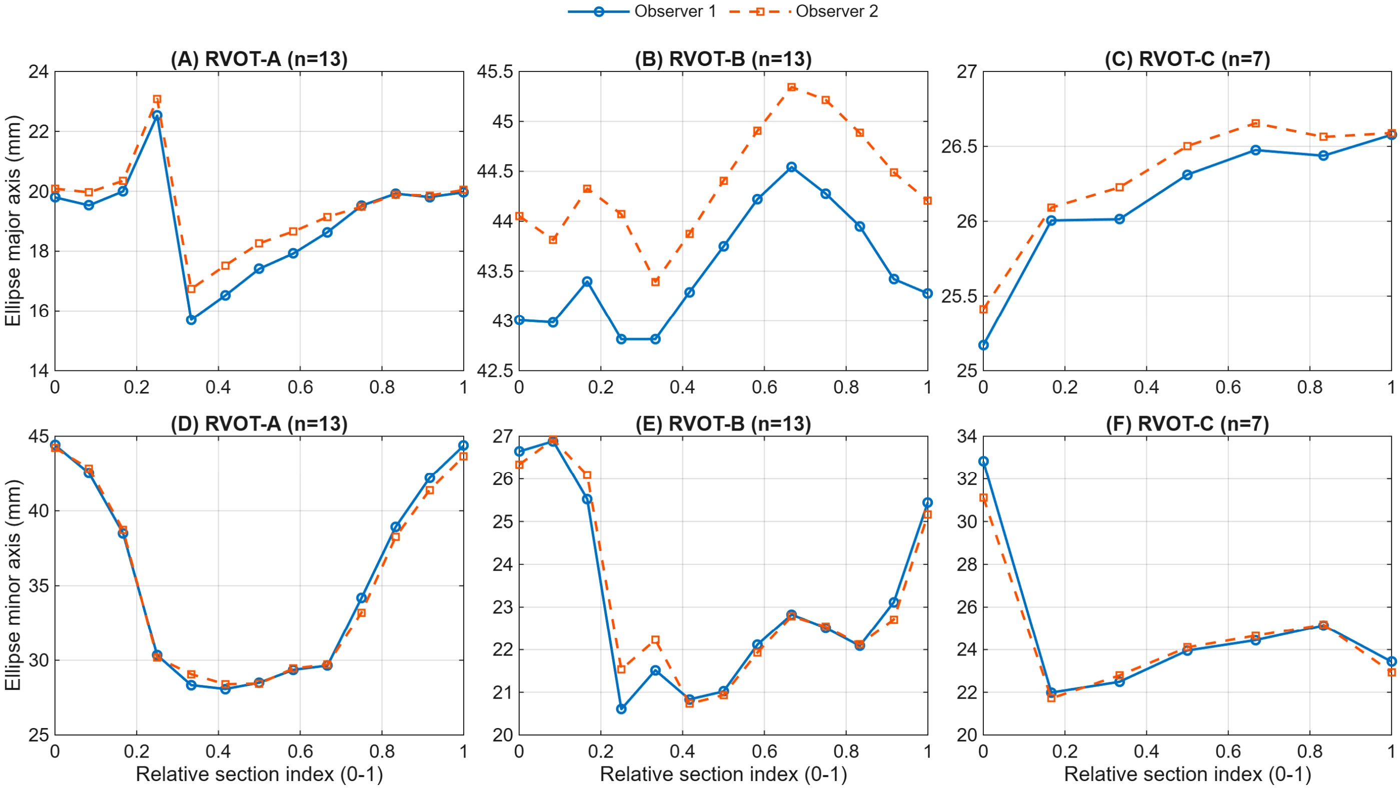

Figure S4. Inter-observer comparison of ellipse-derived dimensions across three RVOT models. Panels (A–C) show the major-axis profiles for RVOT-A, RVOT-B, and RVOT-C, respectively, whereas panels (D–F) present the corresponding minor-axis profiles. Solid blue lines represent Observer 1, and dashed orange lines represent Observer 2. The horizontal axis indicates the normalised sequential index of retained cross-sections (0–1), with 13, 13, and 7 paired sections for RVOT-A, RVOT-B, and RVOT-C, respectively.

Given the small number of independent RVOT models and the use of sequential rather than spatially verified section correspondence, these findings should be interpreted as preliminary evidence of inter-observer agreement in geometric descriptor extraction.

S1.6. Overall interpretation and limitations of the validation

The complementary validation procedures supported the numerical correctness, robustness, and feasibility of the proposed RVOT analysis workflow. The additional exploratory inter-observer assessment demonstrated broadly similar geometric profiles, although local discrepancies remained. However, the limited number of independently analysed models, the absence of intra-observer assessment, and the lack of comprehensive evaluation of operator-dependent processing decisions

preclude definitive conclusions regarding whole-workflow reproducibility. Further validation involving larger datasets and repeated independent processing is warranted.**Table S7. Model-specific numbers of generated, retained and excluded secondary RVOT cross-sections.**

RVOT	Generated	Retained	Excluded	Excluded [%]
1	12	12	0	0.0
2	15	14	1	6.7
3	13	13	0	0.0
4	10	9	1	10.0
5	9	9	0	0.0
6	10	10	0	0.0
7	11	10	1	9.1
8	11	11	0	0.0
9	12	12	0	0.0
10	11	11	0	0.0
11	12	12	0	0.0
12	12	12	0	0.0
13	11	11	0	0.0
14	12	12	0	0.0
15	9	9	0	0.0
16	14	14	0	0.0
17	6	6	0	0.0
18	9	8	1	11.1
19	13	13	0	0.0
20	9	9	0	0.0
21	9	8	1	11.1
22	15	15	0	0.0
23	13	13	0	0.0
24	10	8	2	20.0
25	10	8	2	20.0
26	7	5	2	28.6
27	12	12	0	0.0
28	10	10	0	0.0
29	11	11	0	0.0
30	9	9	0	0.0
31	9	7	2	22.2
32	9	7	2	22.2
33	10	9	1	10.0
34	15	14	1	6.7
35	11	10	1	9.1
36	13	12	1	7.7
37	12	12	0	0.0
38	12	12	0	0.0
39	12	12	0	0.0
40	12	12	0	0.0
41	9	9	0	0.0
42	12	11	1	8.3
43	15	15	0	0.0
44	16	16	0	0.0
45	14	14	0	0.0
46	10	9	1	10.0
47	16	15	1	6.3

48	10	10	0	0.0
49	14	14	0	0.0
50	15	14	1	6.7
51	12	12	0	0.0
52	12	12	0	0.0
53	10	10	0	0.0
54	13	13	0	0.0
55	11	11	0	0.0
56	13	11	2	15.4
57	6	6	0	0.0
58	12	12	0	0.0
59	15	14	1	6.7
60	10	10	0	0.0
Total	687	661	26	3.8
